# Decreased Neuronal Excitability in Medial Prefrontal Cortex during Morphine Withdrawal is associated with enhanced SK channel activity and upregulation of small GTPase Rac1

**DOI:** 10.7150/thno.44893

**Published:** 2020-06-05

**Authors:** Liang Qu, Yuan Wang, Yang Li, Xin Wang, Nan Li, Shunnan Ge, Jing Wang, Gene-Jack Wang, Nora D. Volkow, Bing Lang, Ping Wang, Hao Wu, Jie Zeng, Jian Fu, Jiaming Li, Yue Zhang, Xuelian Wang

**Affiliations:** 1 Department of Neurosurgery, Tangdu Hospital, The Fourth Military Medical University, Xi'an, Shaanxi, 710038, China.; 2 Laboratory of Neuroimaging, National Institute on Alcohol Abuse and Alcoholism, Bethesda, MD, 20892-1013, USA.; 3 National Institute on Drug Abuse, National Institutes of Health, Bethesda, MD, 20892, USA.; 4 School of Medicine, Medical Sciences and Nutrition, Institute of Medical Sciences, University of Aberdeen, Aberdeen, United Kingdom.; 5 National Clinical Research Center for Mental Disorders, Changsha, 410013, China.; 6 Department of Neurosurgery, The First Affiliated Hospital of Xi'an Jiaotong University, Xi'an, 710061, China.; 7 Department of Neurosurgery, Xinjiang Production and Construction Corps Hospital, Xinjiang, 830002, China.

**Keywords:** Morphine, Nucleus Accumbens (NAc), medial prefrontal cortex (mPFC), Small Conductance Calcium-Activated Potassium Channels (SK channels), Rac1

## Abstract

**Rationale:** Neuroadaptations in the medial prefrontal cortex (mPFC) and Nucleus Accumbens (NAc) play a role in the disruption of control-reward circuits in opioid addiction. Small Conductance Calcium-Activated Potassium (SK) channels in the mPFC have been implicated in neuronal excitability changes during morphine withdrawal. However, the mechanism that modulates SK channels during withdrawal is still unknown.

**Methods:** Rats were exposed for one week to daily morphine injections (10 mg·kg^-1^ s.c.) followed by conditional place preference (CPP) assessment. One week after withdrawal, electrophysiological, morphological and molecular biological methods were applied to investigate the effects of morphine on SK channels in mPFC, including infralimbic (IL), prelimbic (PrL) cortices and NAc (core and shell). We verified the hypothesis that Rac1, a member of Rho family of small GTPases, implicated in SK channel regulation, modulate SK channel neuroadaptations during opiate withdrawal.

**Results:** One week after morphine withdrawal, the neuronal excitability of layer 5 pyramidal neurons in IL was decreased, but not in PrL. Whereas, the excitability was increased in NAc-shell, but not in NAc-core. In mPFC, the expression of the SK3 subunit was enhanced after one-week of withdrawal compared to controls. In the IL, Rac1 signaling was increased during withdrawal, and the Rac1 inhibitor NSC23766 disrupted SK current, which increased neuronal firing. Suppression of Rac1 inhibited morphine-induced CPP and expression of SK channels in IL.

**Conclusions:** These findings highlight the potential value of SK channels and the upstream molecule Rac1, which may throw light on the therapeutic mechanism of neuromodulation treatment for opioid dependence.

## Introduction

Opioid use disorders (OUD) are chronic relapsing brain diseases that develop with repeated drug exposure in those that are vulnerable because of genetic, developmental, and/or psychosocial factors 1, 2. The exact mechanisms of OUD are not fully understood, but are generally believed to be related to drug-induced neuroadaptation 3, 4. Several studies have reported a series of relatively long-term opioid-induced molecular biological and neurophysiological changes in brain reward circuit and other regions 5-7. In morphological studies, evidence has suggested that opiate exposure induced neuroadaptations in the mesocorticolimbic system may be critical for addiction-related behavior 8. A better understanding of neurobiological mechanisms of neuroadaptation could lead to more effective treatment strategies for OUD.

The nucleus accumbens (NAc), which receives dopaminergic projections from ventral tegmental area (VTA), plays a crucial role in drug reward 9. Recently, new insights have implied that dopamine neurotransmission is the key projection, but not the only pathway 10-12. Glutamatergic neurotransmission in opioid-related neuronal systems also involved in regulating reward and memory processes 13. Repeated drug-induced activation of brain reward regions can ultimately shape the emergence of drug-related habitual behaviors 14. Previous study indicated that there are individual differences in context-induced response after morphine withdrawal 15. Moreover, following repeated opioid exposures, a reduction in inputs from prefrontal cortex into striatum disrupt the control over action selection, and impair self-regulation and control 16, 17. The medial prefrontal cortex (mPFC), which sends glutamatergic projection to VTA and NAc, is involved with encoding the learned associations between opioid reward and opioid-associated contexts during abstinence 18. In rodents, the prelimbic (PrL) and infralimbic (IL) regions of the mPFC modulate opioid related drug reward, dependence and withdrawal 19-21.

The excitability of neurons in NAc and mPFC can be affected by the amplitude of the action potential afterhyperpolarization (AHP), including both the medium AHP (mAHP), presumed to be regulated by small conductance calcium-activated potassium (SK) channels, and the fast AHP, presumed to be regulated by large conductance calcium-activated potassium channels 22, 23. SK channels impact somatic excitability by contributing to AHP and adjust synaptic plasticity 24. Rodent studies suggest that SK2 channels in IL regulate extinction of drug seeking behavior via mGlu5-mediated facilitation of synaptic plasticity 25. On the other hand, Rac1, a member of Rho family of small GTPases, might modulate SK channel activity and firing patterns 26, 27, and contribute to structural and behavioral plasticity in NAc and mPFC in response to repeated drug exposures 28, 29. However, further exploration is needed to answer the question that whether morphine withdrawal following repeated administration, which induced the enhancement of Rac1 signaling, is directly or indirectly associated with SK channel related structural plasticity. Evidence suggested that molecular neuroadaptations in both the NAc and the lateral dorsal striatum (DStr) could effectively enhance drug-seeking behavior 30, 31. Previous studies demonstrated that drug dependence were established by subcutaneous administration of morphine for more than 7 consecutive days 32, 33. We previously showed enhanced action potential (AP) firing of neurons in the NAc shell after morphine withdrawal was associated with downregulation of SK channels 34. We also reported that neuronal firing in mPFC was decreased whereas SK channels were upregulated after 3 weeks of morphine withdrawal. However, the mechanism underlying the alterations in SK channels with morphine withdrawal is unknown.

In this study, we exposed rats to one-week daily morphine injections, then one week later during withdrawal we applied electrophysiological and a series of behavioral (conditioned place preference), morphological and molecular biological methods to explore the mechanism responsible for the upregulation of SK channels in mPFC. Specifically, we tested the hypothesis that Rac1 signaling after morphine withdrawal modulates SK channels in mPFC and the associated reduced neuronal excitability during morphine withdrawal. Our findings corroborated our hypothesis and identify SK channels as potential therapeutic targets for medication development in OUD.

## Materials and Methods

For detailed materials and methods, please see the **Supplementary Files**. Briefly, we exposed rats to daily morphine injections for one week and studied them after one week of morphine withdrawal. We performed a separate set of experiments including electrophysiology, behavior (CPP), immunohistochemistry, western blotting and retrograde tract-tracing to assess the effects of SK channel related neuro-adaptations in NAc and mPFC (**Table 1 for specifics and samples sizes**). To explore the role of Rac1 in the SK channel alterations, we performed a Rac1 pull-down and detection assay and LC-MS/MS iTRAQ analysis. To confirm the role of Rac1, bilateral injections of lentivirus expressing Rac1 shRNA or control shRNA into the IL were done in animals after one week of morphine withdrawal. Unless otherwise specified, values in results are reported as Means and SEM.

## Results

### NAc shell AP firing is enhanced after one-week morphine withdrawal

**Figure 1:**
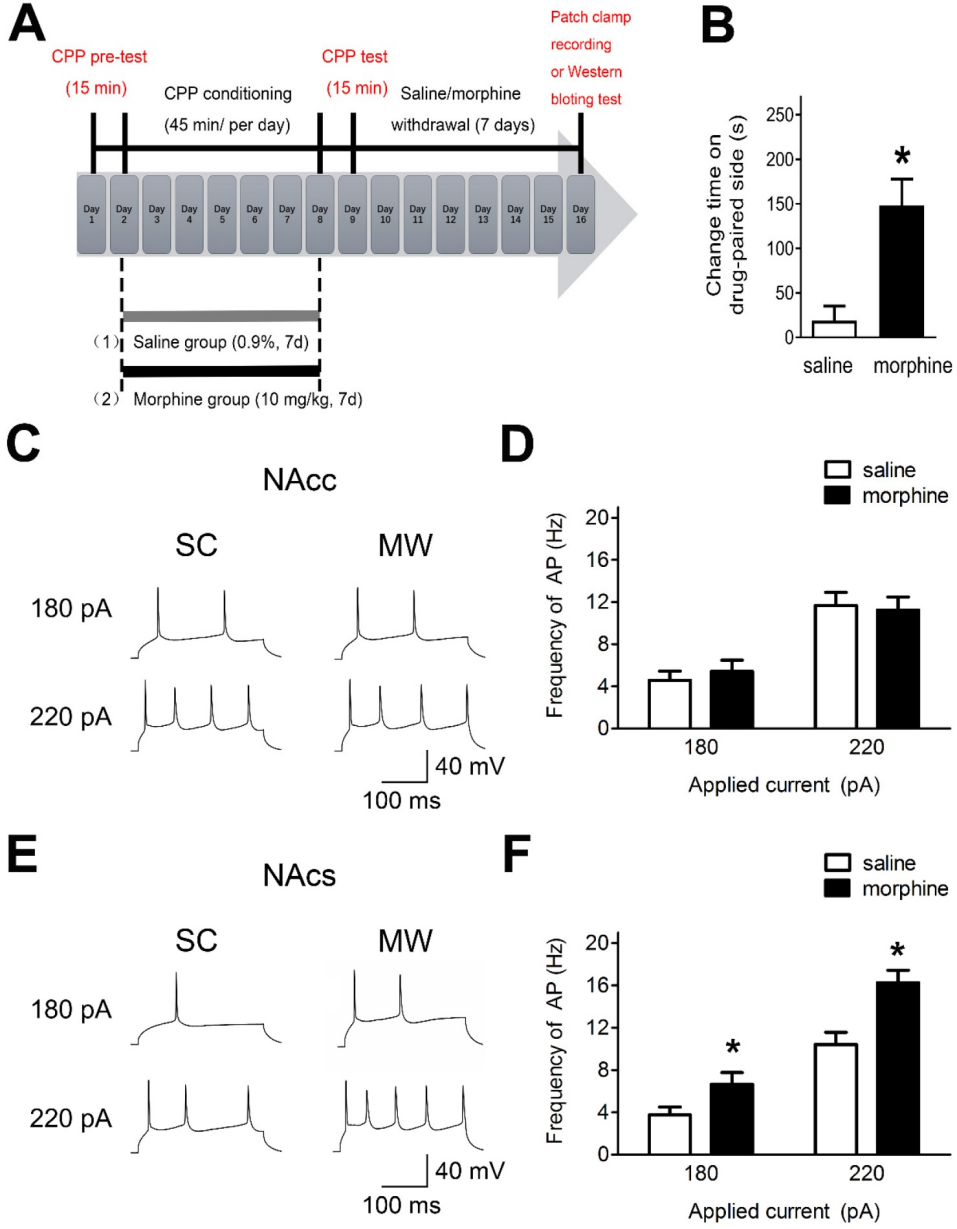
** AP firing in NAc shell was significantly enhanced after one-week morphine withdrawal. A.** Morphine-induced CPP of experimental groups over time. All animals were sacrificed one week after the final CPP test. **B.** For the CPP, the difference in the time spent in the less preferred and drug-paired compartment between the CPP pre-test and CPP test (saline, n=68; morphine, n=71). *P < 0.05 compared with the SC group.** C.** and **E.** Example traced of AP generation evoked in response to depolarizing current steps in NAc core (NAcc) and NAc shell (NAcs) MSNs from SC rats or morphine withdrawal (MW) rats. **D.** Grouped data showing no change of AP firing in NAcc MSNs after one-week MW (NAcc-180pA-saline, n=8; NAcc-180pA-morphine, n=8; NAcc-220pA-saline, n=8; NAcc-220pA-morphine, n=8). **F.** Grouped data showing increased AP firing in NAcs MSNs after one-week MW. NAcc-180pA-saline, n=8; NAcc-180pA-morphine, n=8; NAcc-220pA-saline, n=8; NAcc-220pA-morphine, n=8. Data correspond to means ± S.E.M., *P < 0.05 vs. SC.

To assess the effects of morphine withdrawal on AP firing in NAc core and shell in median spiny neurons (MSNs), we used morphine-induced CPP and performed electrophysiological recordings and western blot analyses (**Figure 1A**). Rats readily acquired morphine-induced CPP and displayed a significant preference for the compartment associated with morphine (10 mg·kg^-1^ s.c.) at the end of 7 days of morphine administration (**Figure 1B**). Electrophysiological recordings were performed after one-week morphine withdrawal in current-clamp mode in NAc core and NAc shell MSNs (**Figure 1C and 1E**), which are hedonic hotspots for reward processing 35, 36.

In NAc shell, MSNs exhibited enhanced AP firing frequency under morphine withdrawal compared with saline mock-treatment control (SC) [**Figure 1D,** 180 pA: NAc shell-MSN-SC, 3.75 ± 0.42 Hz, n(cells) = 12, N(rat) = 8; NAc shell-MSN-MW, 6.66 ± 0.87 Hz, n = 12, N = 8; P = 0.045; 220 pA: NAc shell-MSN-SC, 10.42 ± 1.22 Hz, n(cells) = 12, N(rat) = 8; NAc shell-MSN-MW, 16.25 ± 1.04 Hz, n = 12, N = 8; P = 0.003]. In NAc core MSNs, there was no change in AP firing frequency under morphine withdrawal compared with SC [**Figure 1F,** 180 pA: NAc core-MSN-SC, 4.58 ± 0.64 Hz, n(cells) = 14, N(rat) = 8; NAc core-MSN-morphine withdrawal(MW), 5.41 ± 0.82 Hz, n = 14, N = 8; P = 0.559; 220 pA: NAc core-MSN-SC, 11.66 ± 1.34 Hz, n(cells) = 14, N(rat) = 8; NAc core-MSN-MW, 11.24 ± 1.12 Hz, n = 14, N = 8; P = 0.816]. Thus, after one-week morphine withdrawal, AP firing was increased in NAc shell MSN but not in NAc core MSNs.

### Infralimbic neuronal excitability of layer 5 pyramidal neurons is decreased after one-week morphine withdrawal

**Figure 2:**
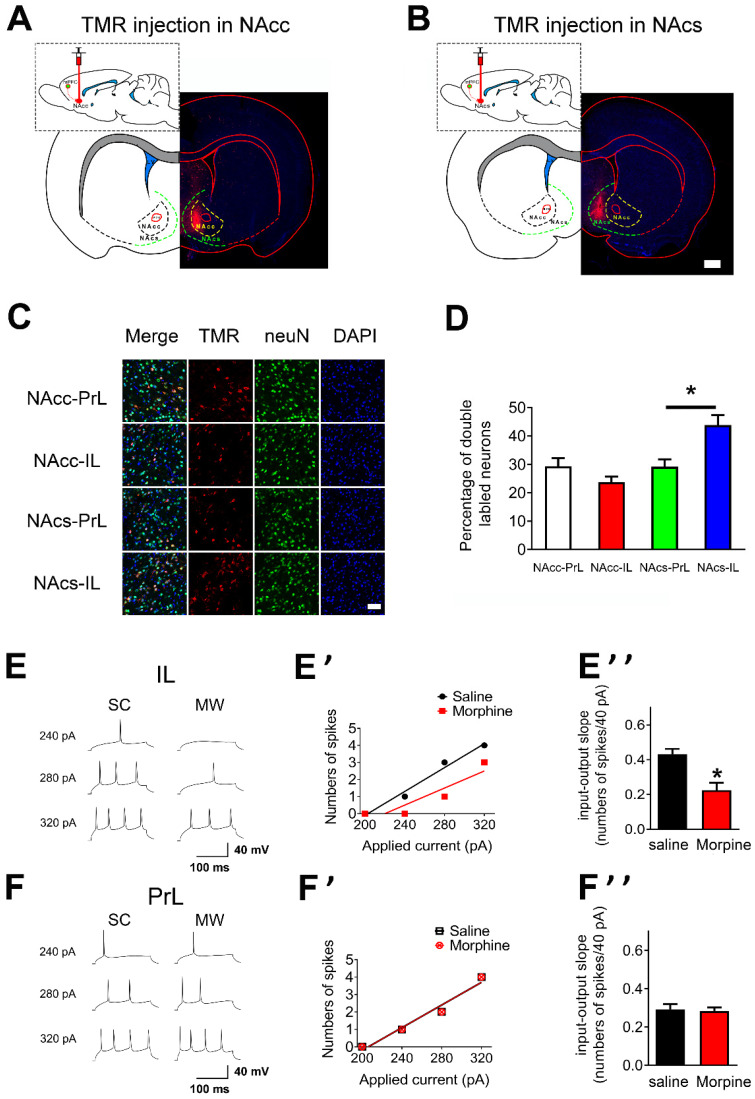
** Neuronal excitability of layer 5 pyramidal neurons in IL is decreased after one-week morphine withdrawal. A.** and **B.** Schematic of the rat brain with subcortical injection (retrograde tracers TMR) into NAcc or NAcs. Scale bar, 1 mm. **C**. Examples illustrating that the triple labeled neurons (TMR, red; neuN, green; DAPI, blue) in “NAcc-PrL”, “NAcc-IL”, “NAcs-PrL”, and “NAcs-IL” projections. Scale bar, 200 μm. **D**. Quantitative analysis of TMR/neuN double labled neurons in “NAcc-PrL”, “NAcc-IL”, “NAcs-PrL”, and “NAcs-IL” projections. (Mean ±S.E.M: NAcc-PrL: n = 15 slice; 29.38±2.85, NAcc-IL: n = 15 slice, 23.75±1.98, NAcs-PrL: n = 18 slice, 29.25±2.59 and NAcs-IL: n = 18 slice, 43.88±3.56 double labeled neurons/100 neurons; p=0.0002 for one-way ANOVA with Bonferroni's Multiple Comparison Test, ^#^P < 0.05 NAcs-IL vs. NAcs-PrL). **E.** and **F.** Example traced of AP generation evoked in response to depolarizing current steps in IL and PrL neurons from SC or MW rats. **E'** and **F'** Example input/output relationships (I/O slope) derived from the SC and MW group traced in **E.** and **F**. **E''** and **F''** Grouped data showing quantitative analysis of I/O slope in IL and PrL neurons from the SC versus MW group. SC, n=14; MW, n=16; Data correspond to means ± S.E.M., *P < 0.05 vs. SC.

To characterize the regional targeting of mPFC's projections to NAc, we first assessed if there were distinct subpopulations of neurons within mPFC that projected distinctly to NAc core and NAc shell 37, 38. To this end, retrograde tracers tetramethylrhodramine-dextran (TMR) were injected into either the NAc core and NAc shell to enable visualization of PrL or IL neurons that projected to each target region (“NAc core-PrL”, “NAc core-IL”, “NAc shell-PrL”, and “NAc shell-IL” subpopulations; **Figures 2A and B**, NAc core: n = 5 rats, NAc shell: n = 6 rats). Most TMR/neuN double labeled neurons were located at layer 5 pyramidal neurons in mPFC (**Figure S1**). The density of NAc core-PrL, NAc core-IL, NAc shell-PrL and NAc shell-IL double labeled neurons differed significantly within layer 5 mPFC (**Figures 2C and D**, NAc core-PrL: 29.38±2.85, NAc core-IL: 23.75±1.98, NAc shell-PrL: 29.25±2.59 and NAc shell-IL: 43.88±3.56 double labeled neurons/100 neurons; p=0.0002 for one-way ANOVA of cell density between the subpopulations). Post hoc *t* test showed that the density of NAc shell-IL double labeled neurons was significantly higher than that of NAc shell-PrL (**Figure 2D**, p= 0.025, Bonferroni's Multiple Comparison Test).

To test whether AP firing in mPFC was altered after one-week morphine withdrawal, we first examined the “input/output slope” (I/O slope) of layer 5 pyramidal neurons in IL and PrL (**Figures 2E, E', F and F'**). The I/O slope was calculated as in our previous study 34. Layer 5 pyramidal neurons in IL from morphine withdrawal rats displayed a significantly smaller basal I/O slope than neurons from SC rats (**Figure 2E''**, SC: n = 12, 0.43 ± 0.03 AP/10 pA; MW: n = 16, 0.23 ± 0.04 AP/10 pA; *t* = 3.924, p = 0.0015, unpaired *t* test), showing that basal neuronal excitability in IL was decreased after morphine withdrawal. In contrast, there was no change in basal I/O slope after morphine withdrawal in PrL (**Figure 2F''**, SC: n = 14, 0.29 ± 0.03 AP/10 pA; MW: n = 16, 0.28 ± 0.03 AP/10 pA; t = 0.226, p = 0.823, unpaired t test). Therefore, one-week morphine withdrawal only altered AP firing in IL pyramidal neurons but not in PrL.

### SK Currents of IL neurons are increased after one-week morphine withdrawal

**Figure 3:**
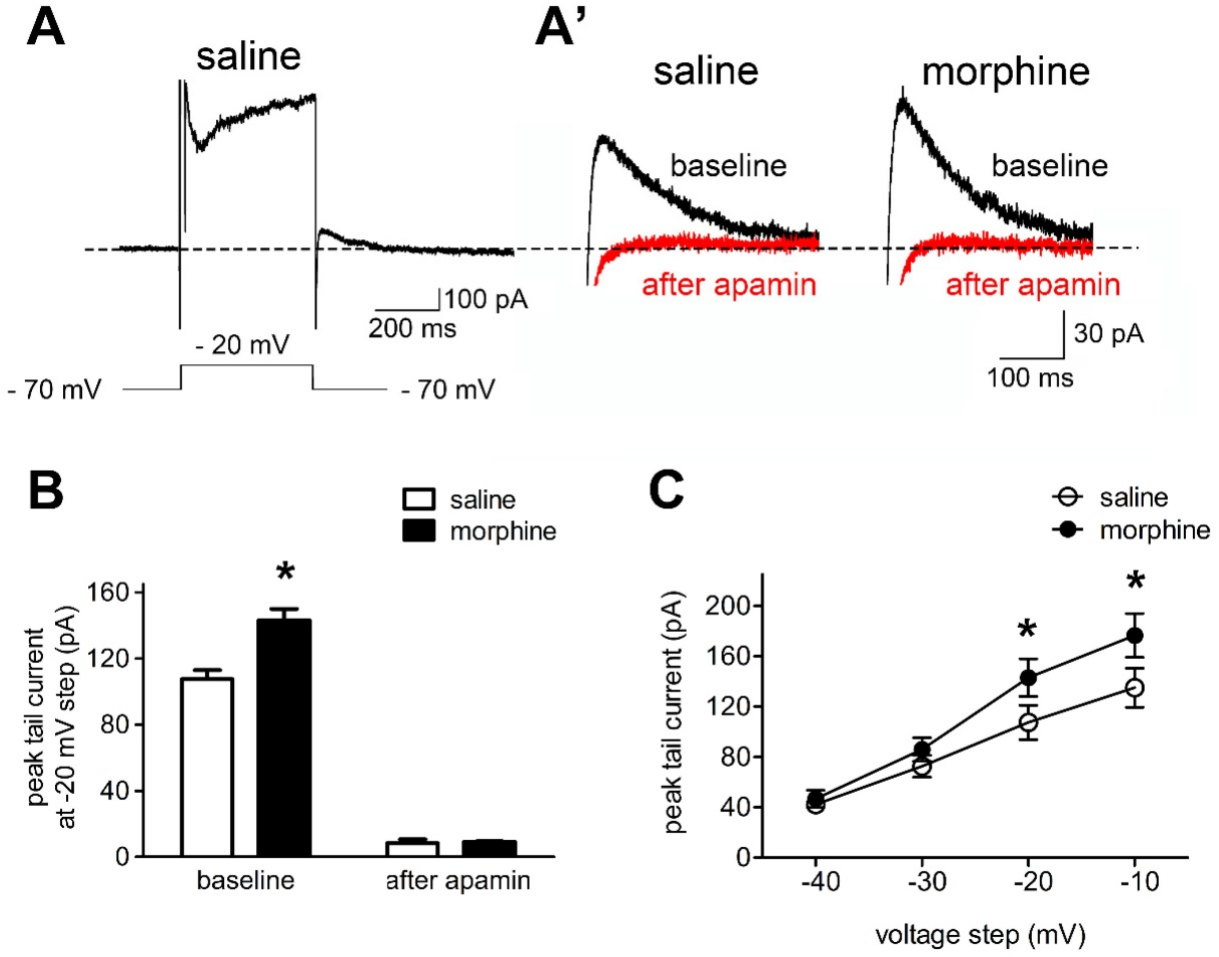
** Morphine withdrawal enhanced SK currents in IL neurons. A.** An example of an entire current response upon depolarization to -20 mV from a -70 mV holding potential in voltage clamp mode, with an apparent tail current after returning to -70 mV following the depolarization; (A') magnified example tail currents.** B.** Grouped data showing that peak tail currents were enhanced in neurons from the morphine withdrawal (MW) versus the SC group (Saline-baseline, n=9; Morphine-baseline, n=8; Saline-after apamin, n=9; Morphine-after apamin, n=8). **C.** Peak tail currents (induced by depolarization to -40 mV ~ -10 mV) were significantly different in neurons from the MW than from the SC group. Saline, n=9; Morphine, n=8. Data correspond to means ± S.E.M., *P < 0.05 *vs.* SC.

In order to test the hypothesis that SK currents contributed to the reduced neuronal basal excitability of IL neurons in morphine withdrawal, we evaluated the effects of SK blockade with apamin on sensitive tail currents in morphine withdrawal and SC rats (**Figures 3A and 3A'**). Peak tail currents after hyperpolarization were extensively larger in IL neurons from morphine withdrawal rats, and apamin approximately eliminated the tail current in both groups (**Figure 3B**; SC group: n = 14 from 9 rats; MW group: n = 12 from 8 rats; baseline: saline 107.5 ± 5.5 pA, morphine 143.0 ± 7.0 pA; after apamin: saline 8.3 ± 2.3 pA, morphine 9.1 ± 0.5 pA; apamin: F_(1,30)_ = 14.20, p = 0.0196; group: F_(1,30)_ = 640.8, p < 0.001; apamin × group: F_(1,30)_ = 15.54, p = 0.0169; two-way RM-ANOVA; *p < 0.05 morphine withdrawal group versus SC group before apamin). This suggested that the peak tail current predominantly reflected SK-mediated currents. Enhanced basal tail currents in neurons from morphine withdrawal versus SC were also evident in Current-Voltage relationships, including those not tested with apamin. Average basal tail current peak amplitudes at voltages from -40 mV to -10 mv in the SC group were 41.2 ± 4.8 pA, 72.6 ± 7.9 pA, 107.5 ± 13.2 pA, 135.0 ± 15.1 pA, and those in the morphine withdrawal group were 46.7 ± 6.4 pA, 85.9 ± 9.4 pA, 143.0 ± 14.9 pA and 176.5 ± 17.5 pA, respectively (**Figure 3C**; n = 9 for SC, n = 8 for MW; voltage: F_(3,60)_ = 232.87, p < 0.001; group: F_(3,60)_ = 4.126, p < 0.001; voltage × group: F_(9,60)_ = 3.164, p < 0.001; two-way RM-ANOVA; *p < 0.05 MW versus SC). These results indicate that morphine withdrawal-induced increases in SK currents contribute to the decreased excitability of IL neurons.

### SK3 subunit protein expression of mPFC is enhanced after one-week morphine withdrawal

**Figure 4:**
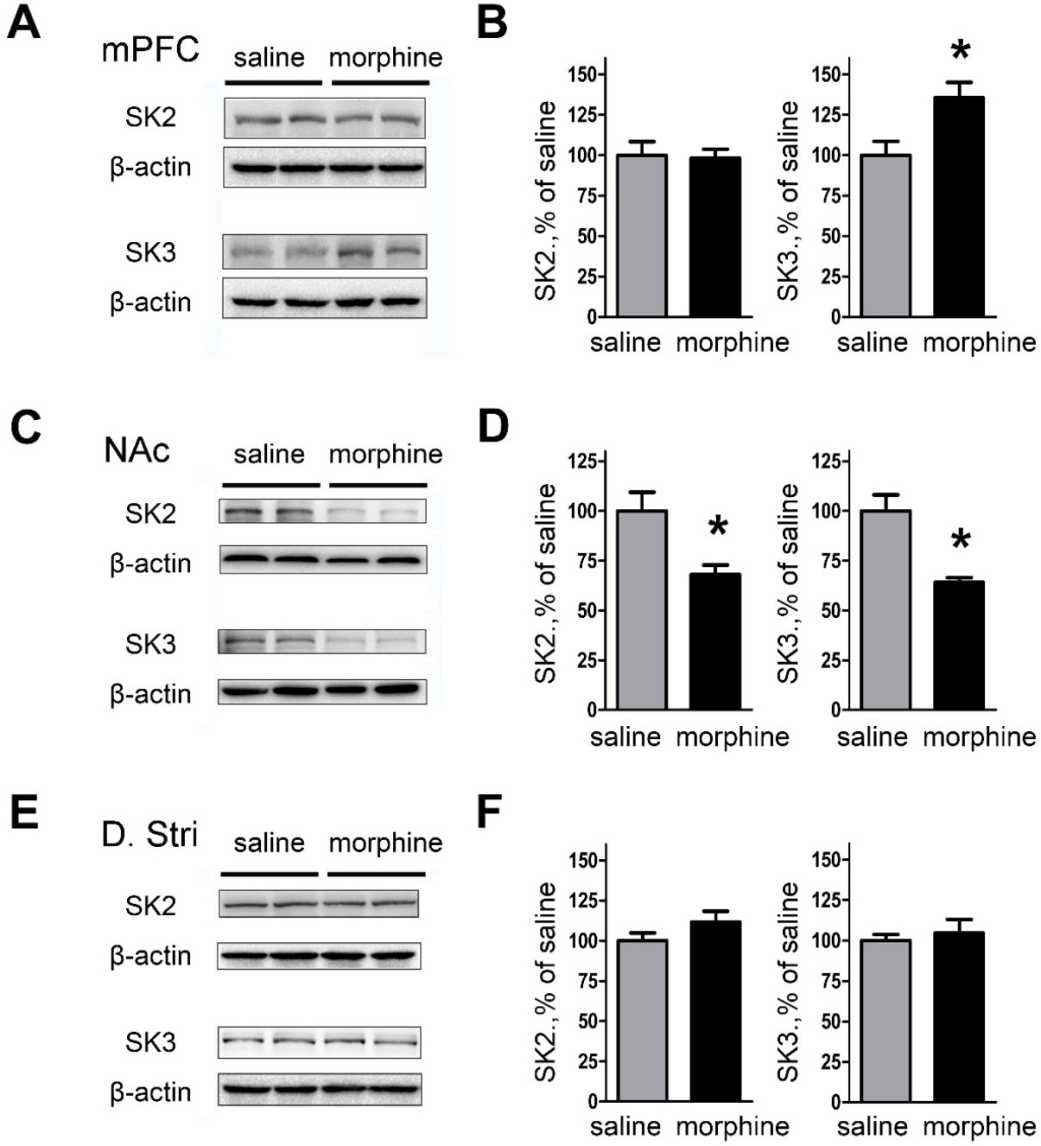
** Protein expression of SK2 and SK3 subunits was changed in NAc and mPFC after one-week morphine withdrawal. A.** Representative western blotting shows the changes of SK2 and SK3 subunit protein expression in mPFC after MW. **B.** Quantitative analysis of SK2 and SK3 subunit protein expression in** A**, normalized to β-actin (SK2: saline, n=7; morphine, n=7. SK3: saline, n=8; morphine, n=8).** C.** Representative western blotting showed the changes of SK2 and SK3 subunit protein expression in NAc after MW. **D.** Quantitative analysis of SK2 and SK3 subunit protein expression in** C**, normalized to β-actin (SK2: saline, n=6; morphine, n=6.SK3: saline, n=6; morphine, n=6).** E.** Representative western blotting showed the changes of SK2 and SK3 subunit protein expression in dorsal striatum after MW. **F.** Quantitative analysis of SK2 and SK3 subunit protein expression in** E**, normalized to β-actin. The data correspond to means ± S.E.M., *P < 0.05 *vs.* SC.

We next examined the effect of morphine withdrawal on SK2 and SK3 channel expression to determine if the increase in peak tail currents was due to channel upregulation. The results showed that SK3 subunit expression was significantly increased in mPFC (both IL and PrL) of morphine withdrawal compared to SC rats (**Figures 4A and 4B**; SK2: saline, 99.99 ± 8.45%; morphine, 98.08 ± 5.66%; t_(12)_ = 0.1878, p = 0.856; SK3: saline, 100.0 ± 8.52%; morphine, 135.4 ± 9.61%; t_(14)_ = 3.050, p = 0.033, unpaired t test, *p < 0.05). In contrast, the expression of SK2 and SK3 was significantly decreased in NAc core and NAc shell of morphine withdrawal rats compared to SC rats (**Figures 4C and D**; SK2: saline, 100.0 ± 9.32 %; morphine, 68.01 ± 4.78 %; t_(10)_ =3.056, p = 0.022; SK3: saline, 99.98 ± 8.06%; morphine, 63.99 ± 2.56 %; t_(10)_ = 4.257, p = 0.005, unpaired t test, *p < 0.05). In addition, no changes in expression of SK2 or SK3 subunits were observed in DStr (**Figures 4E and 4F;** SK2: saline, 99.98 ± 4.79 %; morphine, 111.6 ± 6.76%; t_(14)_ = 1.403, p = 0.210; SK3: saline, 100.0 ± 3.72%; morphine, 104.5 ± 8.50%; t_(14)_ = 0.482, p = 0.647, unpaired t test, *p < 0.05). Our results show that the expression of SK3 in mPFC after one week morphine withdrawal was enhanced.

### PP2A and CK2 are regulated in mPFC and NAc after morphine withdrawal

Because the altered SK currents were associated with changes in SK expression, we assessed whether the expression of the phosphorylating kinase CK2 and the dephosphorylating phosphatase PP2A, subunits of SK were modified after morphine withdrawal in mPFC and NAc. We also further assessed if there were changes in the activity of PP2A.

**Figure 5:**
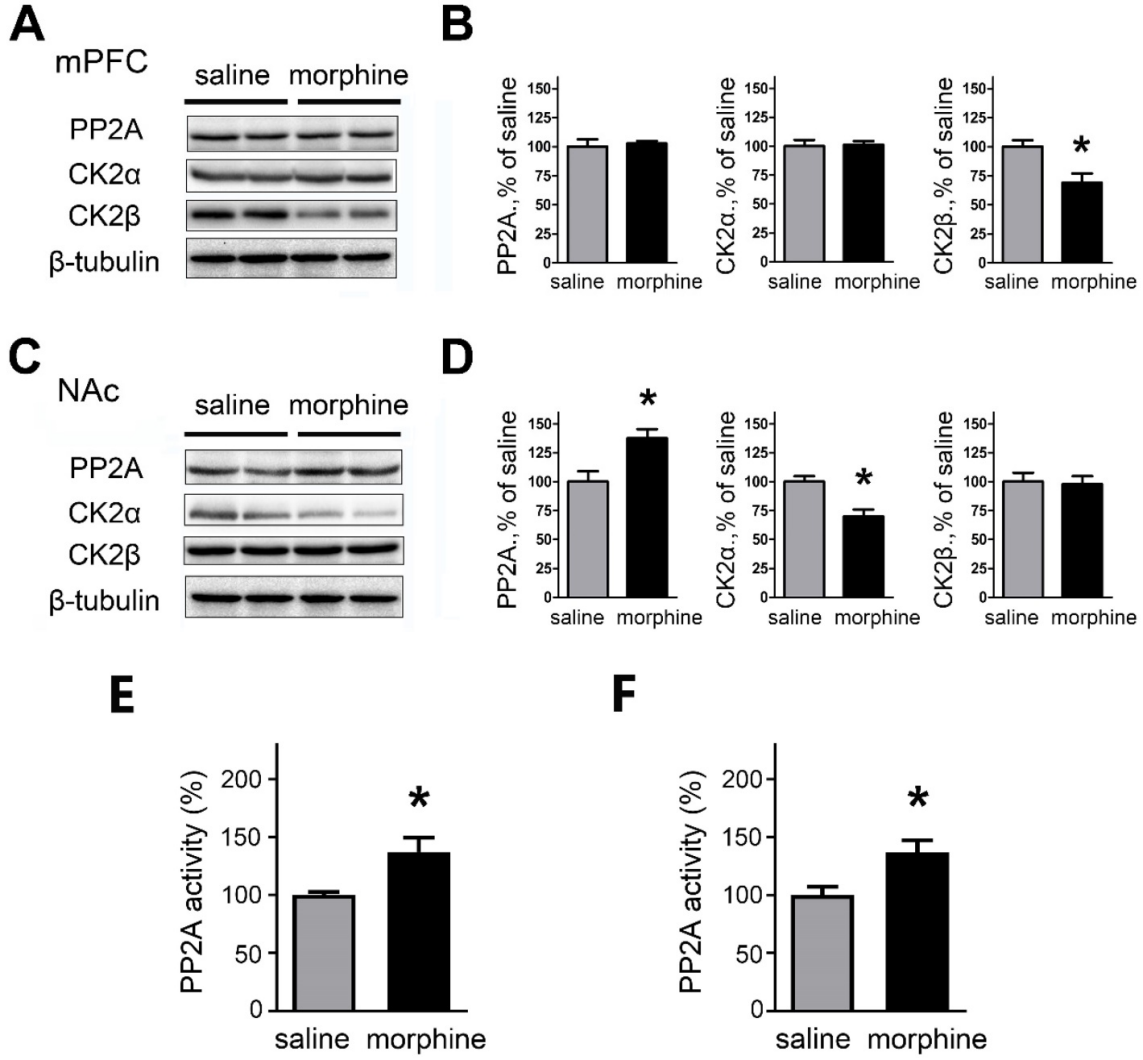
** PP2A and CK2 were regulated in mPFC and NAc after one-week morphine withdrawal. A.** Representative western blotting showed the changes of PP2A, CK2α and CK2β protein expression in mPFC after MW. **B.** Quantitative analysis of PP2A, CK2α and CK2β protein expression in** A**, normalized to β-tubulin (saline, n=7; morphine, n=7).** C.** Representative western blotting showed the changes of PP2A, CK2α and CK2β protein expression in NAc after MW. **D.** Quantitative analysis of PP2A, CK2α and CK2β protein expression in** C**, normalized to β-tubulin (saline, n=8; morphine, n=8). *P < 0.05 *vs.* SC. **E.** PP2A activity was increased in mPFC after one-week MW. **F.** PP2A activity was increased in NAc after one-week MW. Data correspond to means ± S.E.M., *P < 0.05 *vs.* SC.

In the mPFC, western blots and quantification indicate that one-week morphine withdrawal did not affect PP2A and CK2α (PP2A: saline, 99.99 ± 6.24 %; morphine, 102.7 ± 2.20%; t_(12)_ = 0.414, p = 0.693; CK2α: Mean ±S.E.M; saline, 99.98 ± 5.48%; morphine, 101.2 ± 3.12%; t_(12)_ = 0.205, p =0.843, unpaired t test); whereas CK2β was downregulated in morphine withdrawal compared to SC rats (saline, 100.0 ± 5.54%; morphine, 68.86 ± 8.06%; t_(12)_ = 3.185, p =0.019, unpaired t test, *p < 0.05 ) (**Figures 5A and 5B**). In NAc, the expression of PP2A was upregulated (saline, 100.0 ± 8.98%; morphine,137.5 ± 7.85%; t_(14)_ = 3.147, p =0.020, unpaired t test, *p < 0.05) and CK2α was downregulated (saline, 99.97± 4.83%; morphine, 69.92 ± 5.92%; t_(14)_ = 3.935, p =0.008, unpaired t test, *p < 0.05 MW versus SC in NAc) but there were no differences in expression of CK2β in morphine withdrawal compared to SC rats (saline, 100.0 ± 7.66%; morphine, 97.85 ± 6.98%; t_(14)_ = 0.208, p =0.842, unpaired t test) (**Figures 5C and 5D**). We also measured the activity of PP2A after morphine withdrawal in mPFC and NAc (**Figures 5E and 5F**) using the PP2A Colorimetric Assay kit. In mPFC, the activity of PP2A was increased after morphine withdrawal, consistent with the increased SK currents (**Figure 5E**, saline, 100.0 ± 2.66%; morphine, 137.2 ± 12.21%; t_(10)_ = 2.975, p =0.025, unpaired t test). In NAc, the activity of PP2A was also increased (**Figure 5F**, saline, 100.0 ± 7.09%; morphine, 136.7 ± 10.47%; t_(10)_ = 2.906, p =0.027, unpaired t test) consistent with the increased expression of PP2A protein.

### Rac1 activity is increased in the mPFC after one-week morphine withdrawal

**Figure 6:**
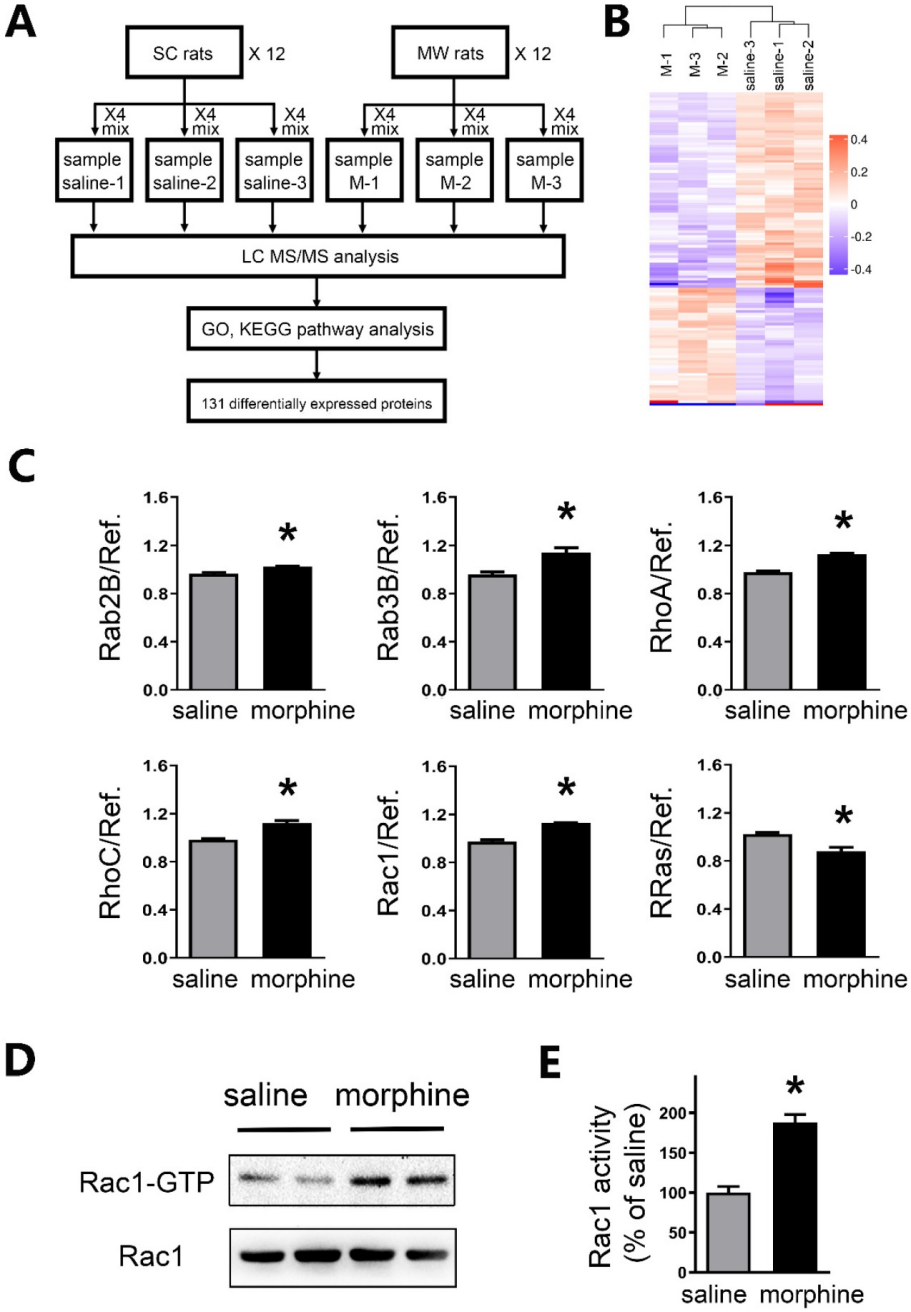
** Rac1 activity was enhanced in mPFC after one-week morphine withdrawal. A.** Diagram of experimental design. Six iTRAQ labels were used: sample saline-1 (n=4), sample saline-2 (n=4) and sample saline-3 (n=4) for the saline-1, saline-2 and saline-3; sample morphine-1 (n=4), sample morphine-2 (n=4) and sample morphine-3 (n=4) for the M-1, M-2 and M-3 respectively. Bioinformatics analysis (GO, KEGG Pathway, etc) was used to examine 131 differential expression proteins. **B.** Global protein expression patterns in each group. K-means clustering representation of total DEP profiles. The magnitude of the percentage is represented by a color scale going from low (blue) to high (red). **C.** Protein expression of altered small GTPases in morphine withdrawal rats compared with SC rats. *P < 0.05 *vs.* SC. **D.** MW resulted in activation of Rac1 (Rac1-GTP). **E** Quantitative analysis of Rac1-GTP in **D**, normalized to Rac1. Data correspond to means ± S.E.M., *P < 0.05 vs. SC.

To further investigate the mechanism of protein level changes, brain tissue in mPFC underwent high accuracy LC-MS/MS iTRAQ analysis (**Figure 6A**). To reduce individual variation, 12 SC rats were pooled into three samples as saline1, saline2, and saline3. While 12 morphine withdrawal rats were pooled into three samples as M1, M2 and M3. Within both the morphine withdrawal and SC groups, a total of 7,187 proteins were identified with at least one unique peptide and a 1% false discovery rate. According to the criteria of p value <0.05 and fold changes >1.10 or <0.91, 131 proteins exhibited significant differential expression between the two groups (**Figure 6B**). Of these, 51 proteins were upregulated, and 80 proteins were downregulated in the morphine withdrawal group compared with the SC group. Then, all differentially expressed proteins were annotated by GO analysis and functional classification (**Figure S2**).

Importantly, LC-MS/MS iTRAQ data showed that six members of small GTPases in RAS superfamily were altered, including five upregulated (Rab2B, Rab3B, RhoA, RhoC and Rac1) and one downregulated (R-Ras) (**Figure 6C and Table S2**). Previous studies suggested that the intensities of active Rac1 (Rac1-GTP) was increased in the IL at a few hours after morphine withdrawal 29. By using Rac1 pull-down and detection assay, we further assessed the activity of small GTPase Rac1 in the mPFC after one-week morphine withdrawal. As shown in **Figures 6D and 6E**, a significant increase in the levels of Rac1-GTP (active Rac1) was detected in the mPFC (saline, 100.0 ± 7.66%; morphine, 187.9 ± 9.92%; *t* = 7.008, p = 0.0004, unpaired *t* test, *p < 0.05 MW versus SC).

### Rac1 inhibitor NSC23766 disrupts SK current and firing regularity in IL neurons

**Figure 7:**
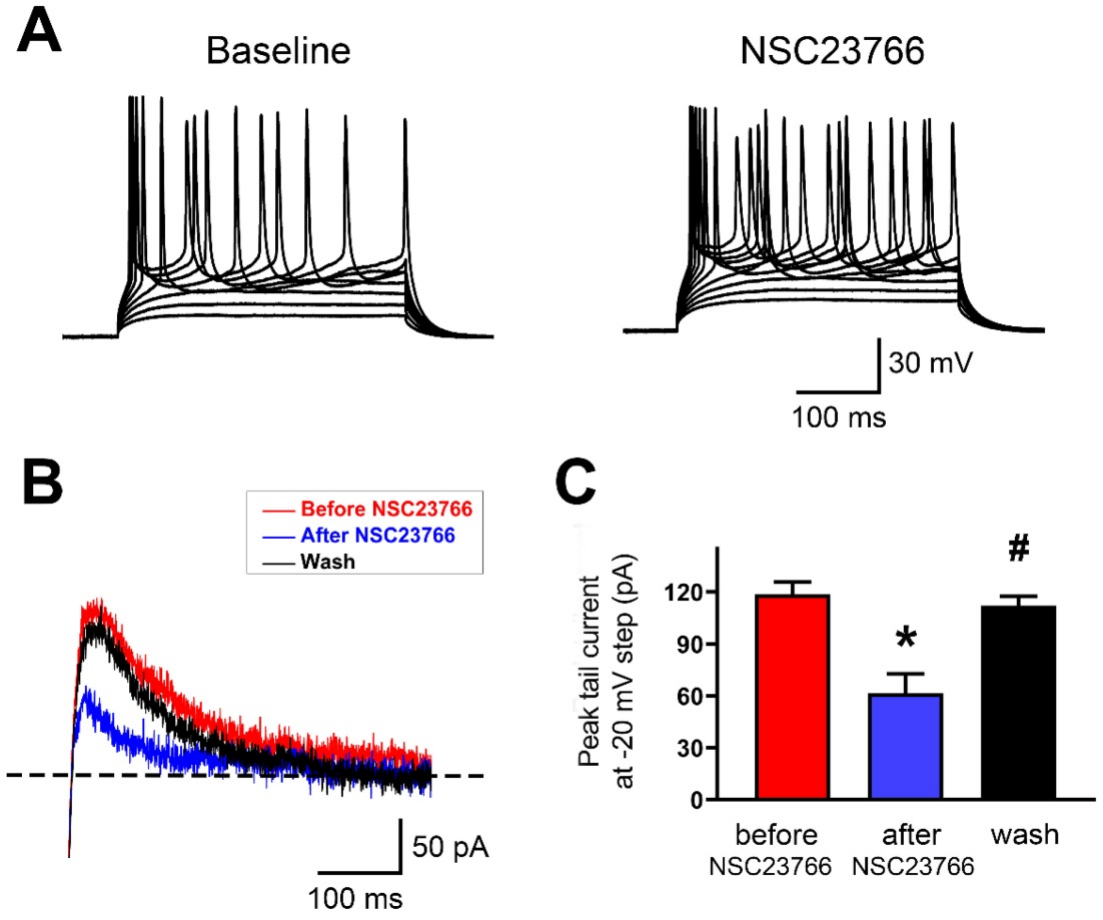
** SK channel activity was regulated by Rac1 in IL neurons. A.** Representative response of step currents (180 pA~340 pA. Δ=20 pA) induced AP firing to bath application of NSC23766 (100 μM) in layer 5 pyramidal neurons of IL. **B.** Representative traces of SK-mediated tail currents in IL neurons before application of 100 M NSC23766 (red), during (blue) bath application of 100 M NSC23766 and wash (black). **C.** Quantitative analysis of SK-mediated tail currents in **B**. Baseline, n=9; After NSC23766, n=9; Wash, n=9. Data correspond to means ± S.E.M., *P < 0.05 vs. Baseline, ^#^P < 0.05 vs. After NSC23766.

To test whether acute blockade of Rac1 alters AP firing and SK currents in layer 5 pyramidal neurons of IL, we used whole-cell current clamp recordings to monitor firing pattern changes and SK-mediated tail currents in response to Rac1 blockade by NSC23766 (100 μM). Bath perfusion of neurons with NSC23766 dramatically increased their firing frequency and induced them to fire irregularly (**Figure 7A;** 13 neurons in 9 rats were tested). Next, we measured SK-mediated peak tail current before and after NSC23766. In layer 5 pyramidal neurons of IL, NSC23766 reduced the peak tail current (**Figures 7B and 7C**; Baseline: 118.5 ± 2.91 pA, After NSC23766: 61.34 ± 4.61 pA and Wash: 111.9 ± 2.25 pA; p < 0.0001 for one-way ANOVA followed by Bonferroni's Multiple Comparison Test; Baseline vs. After NSC23766, *p < 0.001; After NSC23766 vs. Wash, ^#^p < 0.001; Baseline vs. Wash, p = 0.383), which was consistent with SK inhibition enhanced neuronal firing. All these results show that Rac1 signaling plays crucial role in regulating function of SK channels in the IL.

### Genetically downregulating Rac1 activity in the IL regulates morphine-induced CPP and expression of SK subunit channels

The above results suggest that Rac1 activity was involved in regulating AP firing and SK channel activity in IL. Thus, to confirm the role of Rac1 activity in SK channel expression and morphine-induced CPP, we genetically manipulated Rac1 levels within the IL.

**Figure 8:**
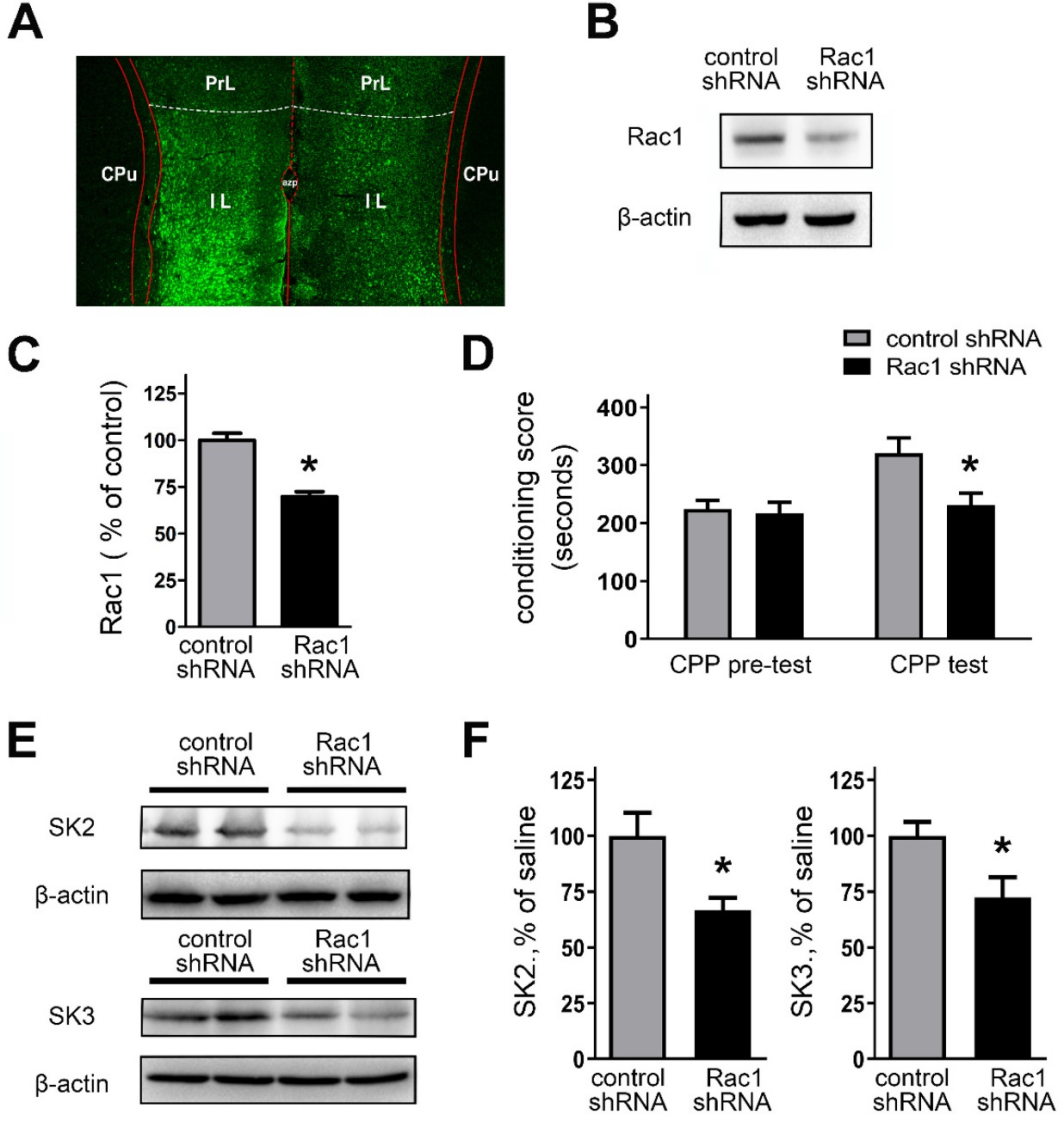
** Knockdown of the Rac1 expression by Rac1-shRNA impaired CPP behaviors and suppressed expression of SK channels. A.** Infection of the IL with lentivirus expressing Rac1-shRNA was visualized by fluorescence microscope. **B.** Infection of lentivirus expressing Rac1-shRNA attenuated Rac1 expression within the IL. **C.** Quantitative analysis of Rac1-GTP in **B**, normalized to β-actin. Control shRNA, n=8; Rac1 shRNA, n=10. *P < 0.05 vs. control shRNA. **D.** Knockdown of the Rac1 expression in the IL with Rac1-shRNA impaired CPP behaviors. **E.** and** F.** Knockdown of the Rac1 expression in the IL with Rac1-shRNA suppressed expression of SK channels subunits. Control shRNA, n=6; Rac1 shRNA, n=6. Data correspond to means ± S.E.M., *P < 0.05 vs. control shRNA.

We assessed the validity of *in vivo* knockdown of Rac1 expression using Rac1 shRNA (**Figures 8A and 8B**). Bilateral stereotactic injections of lentivirus, expressing Rac1 shRNA or control shRNA, were administrated into the IL. Next, the rats received CPP training after two weeks. **Figure 8A** revealed that bilateral IL injections of lentivirus caused effective infection (tagged GFP staining) with Rac1 shRNA in the IL. As shown in **Figures 8B and 8C**, Rac1 shRNA-expressed rats showed a significant decrease of Rac1 protein expression, compared with the control shRNA group (control shRNA, 100.0 ± 3.72%; Rac1 shRNA, 69.72 ± 2.78%; t_(16)_ = 6.524, p =0.0006, unpaired t test, *p < 0.05). We also examined the impact of Rac1-shRNA prior to morphine exposure on morphine-induced CPP. Rac1-shRNA group significantly reduced morphine-induced CPP (**Figure 8D**; main effect of gene: F_(1,28)_ = 5.432, *p* = 0.0272, test: F_(1,28)_ = 7.118, p = 0.0125; main effect of gene x test: F_(1,28)_ = 3.988, p = 0.0556; two-way RM-ANOVA, *p < 0.05 compared with control shRNA). And we further observe the locomotor activity followed by genetically downregulating Rac1 activity (**Table 2**). The data showed that morphine-withdrawal rats with Rac1 shRNA exhibited a greater level of inhibition on locomotor activity compared to morphine-withdrawal rats with Rac1 shRNA.

Moreover, Rac1-shRNA-expressed rats also revealed a significant decrease in the expression of SK2 or SK3 channel subunits (**Figures 8E and 8F**; SK2: control shRNA, 100.0 ± 10.24%; Rac1 shRNA, 66.48 ± 5.65%; t_(10)_ = 2.867, p = 0.0168, unpaired *t* test, *p < 0.05; SK3: control shRNA, 100.0 ± 6.22%; Rac1 shRNA, 72.22 ± 9.29%; t_(10)_ = 2.485, p = 0.0323, unpaired *t* test, *p < 0.05). To further confirm whether Rac1 plays a role in regulating the protein expression level of SK2 and SK3 channel subunits, Rac1 in primarily cultured cortical neurons was knocked down by employing AAV expressing Rac1-shRNA, and then rescued by AAV expressing Rac1 (**Figures S3**). All these results show that Rac1 within the IL is essential for morphine-induced CPP and regulation of SK channel subunits.

## Discussion

In the present study, we show distinct neuroadaptation in mPFC than in NAc after one-week morphine withdrawal following CPP to morphine (one-week exposure 10 mg·kg^-1^ s.c.), including reduced neuronal excitability in mPFC and increased excitability in NAc core. We also provide evidence that changes in SK subunit expression and activity are involved in these changes. Specifically, our results demonstrate that in rats studies one-week after morphine withdrawal: (1) the excitability of pyramidal neurons in layer 5 of IL was decreased, whereas that of MSN in the NAc shell was increased; (2) in mPFC, SK currents and the expression of SK subunit SK3 were increased, whereas in NAc the expression of SK2 and SK3 were decreased; (3) the expression of the SK subunits, PP2A, CK2α and CK2β were differentially regulated in mPFC and NAc though the activity of PP2A was increased both in mPFC and NAc; (4) Rac1 expression and signaling was increased in IL and its blockade (*NSC23766*) increased their firings and reduced SK currents; and (5) knockdown of Rac1 expression using Rac1 shRNA reduced morphine-induced CPP and reduced the expression of SK2 and SK3. Our findings indicated that decreased neuronal excitability in mPFC and enhanced excitability in NAc are consistent with our prior studies, which focused on rats with three weeks after morphine withdrawal 34. This indicates that our findings are robust and that the neuronal changes in mPFC and NAc occur both at one and three weeks after morphine withdrawal. Our findings also implicate Rac1 inhibition could increase neuronal firing by modulating SK activity and its expression in IL. This provides preliminary evidence that Rac1 signaling contribute to morphine-induced CPP.

Opioid withdrawal is characterized by somatic and psychological symptoms, and both symptoms emerge as a result of neuroadaptation in neural circuits 39. The mPFC plays a crucial role in regulating the activity of the NAc, including that associated with drug craving and drug withdrawal 29, 34. Moreover, there is evidence of a top-down circuit that controls reward from PFC to NAc 8, 40. The NAc is comprised of the NAc core and shell sub-regions, which have distinct connectivity and dissociable roles in their responses to drugs and in conditioning 41. The NAc core appears to be involved in drug cue memories, drug-seeking behavior and coordinating behavioral output 42. The NAc shell interacts with limbic and autonomic brain regions and is therefore believed to be involved in the rewarding, emotional, and visceral responses to reinforcing stimuli 43. Within NAc core or shell, D1-MSNs and D2-MSNs both receive excitatory glutamate afferents, but play different roles in reward circuit 44, 45. Our present study shows that morphine withdrawal could decrease SK channel mediated neuronal excitability in IL-mPFC and enhance neuronal excitability in NAc shell. Future studies are needed to further assess differential neuroadaptations to morphine withdrawal between the D1-MSNs and the D2-MSNs in NAc core and shell.

In rodents, the mPFC can be functionally divided into prelimbic cortex (dorsal subregion) and infralimbic cortex (ventral subregion) 46. The PrL division has multiple functions, such as action-selection, instrumental learning and drug seeking, goal-directed behavior, and extinction 47. Glutamatergic projections from the PrL and the basolateral amygdala (BLA) to the NAc core are known to drive drug-seeking behavior 15, 48. PrL lesions or inactivation produces deficits in attentional selectivity and increases in perseverative responding 49, 50. The role of the IL is controversial. It appears to play a role in behavioral inhibition and may be more involved in habitual responding than in goal-directed behavior 51. Previous studies showed that IL might indirectly inhibit outputs to the NAc core. In addition, the IL projection to the NAc shell exerts inhibitory regulation over drug seeking and its inactivation was shown to increase heroin seeking behavior 52. During morphine withdrawal (**Figures 1F and 2E''**), our current findings are consistent with a modulatory role of the IL projection to NAc shell for inhibition of reward, which is in agreement with our previous findings 34.

These regional neuroadaptations may indicate differential impairments in the molecular mechanism regulating the mPFC-NAc control-reward circuit. Numerous studies have provided evidence that SK channels contribute to adaptive plasticity of glutamatergic synapses and neuronal excitability 34, 53, 54. Prior research demonstrated that positive modulation of SK channels in IL attenuated mGlu5-dependent facilitation of cue-conditioned extinction learning 25. Our study revealed that SK3 subunit expression was enhanced in mPFC after morphine withdrawal (**Figure 4B**). We further confirmed that the activity of SK channels was enhanced in layer 5 pyramidal neurons of IL (**Figures 3B and C**). Previous studies implied that actions of the intracellular CK2 and PP2A on the SK-calmodulin (CaM) complex could regulate the Ca^2+^ sensitivity of SK channels 55. Our results revealed that the expression level of PP2A, CK2α and CK2β were differently altered in mPFC and NAc after morphine withdrawal (**Figures 5B and D**). In addition, PP2A activity, which exerts a complementary control role on SK channels, was enhanced in mPFC and NAc after morphine withdrawal (**Figures 5E and F**). Our findings confirmed the presence of altered SK channels in the mPFC-NAc circuit. Furthermore, these observations provide evidence that morphine withdrawal induced neuronal firing decrease in IL are mediated in part by neuroadaptations in SK channel activity in mPFC. However, the mechanism underlying the differential changes in the expression of SK channels during morphine withdrawal between NAc and mPFC still needs further investigation.

The small GTPases from the Ras superfamily play crucial roles in basic cellular processes, including drug associated reward, abstinence and advanced executive function 56. Rac1, a member of Rho Subfamily of small GTPases, regulates key cellular functions in the central nervous system 28. Previously a study suggested that Rho signaling was involved in regulating neuronal synaptic plasticity in the NAc and VTA 57, and that Rac1-dependent GABA-A receptor endocytosis played a crucial role in the extinction of aversive memories 29. In our study, we applied high accuracy LC-MS/MS iTRAQ to analyze the differential protein expression in mPFC between the morphine withdrawal and SC groups. We observed five up-regulated small GTPases (including Rac1, Rab2B, Rab3B, RhoA and RhoC) and one down-regulated (R-Ras) in mPFC after morphine withdrawal (**Figure 6C**). We further showed that the active Rac1 (Rac1-GTP) was enhanced in mPFC after morphine withdrawal. In addition, we provide preliminary evidence of quantitative differences in expression of Rab2B and Rab3B in mPFC. Studies have reported that the protein level of Rac1 was increased in addicted rats and in rats undergoing drug withdrawal 29, 58, 59. In addition, SK channel activity was regulated by Rac1 inhibition in cerebellar neurons 26. Locomotor activity is considered to be a behavioral response of a hypersensitive reward pathway after morphine withdrawal 60. In our present study, morphine-withdrawal rats with Rac1 shRNA exhibited a greater level of inhibition on locomotor activity compared to morphine-withdrawal rats with Rac1 shRNA (**Table 2**). We interpret our results as indicating that enhanced Rac1 signaling regulates the increased activity of SK channels, which in turn contributes to the decreased excitability of IL-mPFC neurons. And both reward response and withdrawal response would be affected by genetically downregulating Rac1 in the IL cortex.

## Conclusion

**Figure 9:**
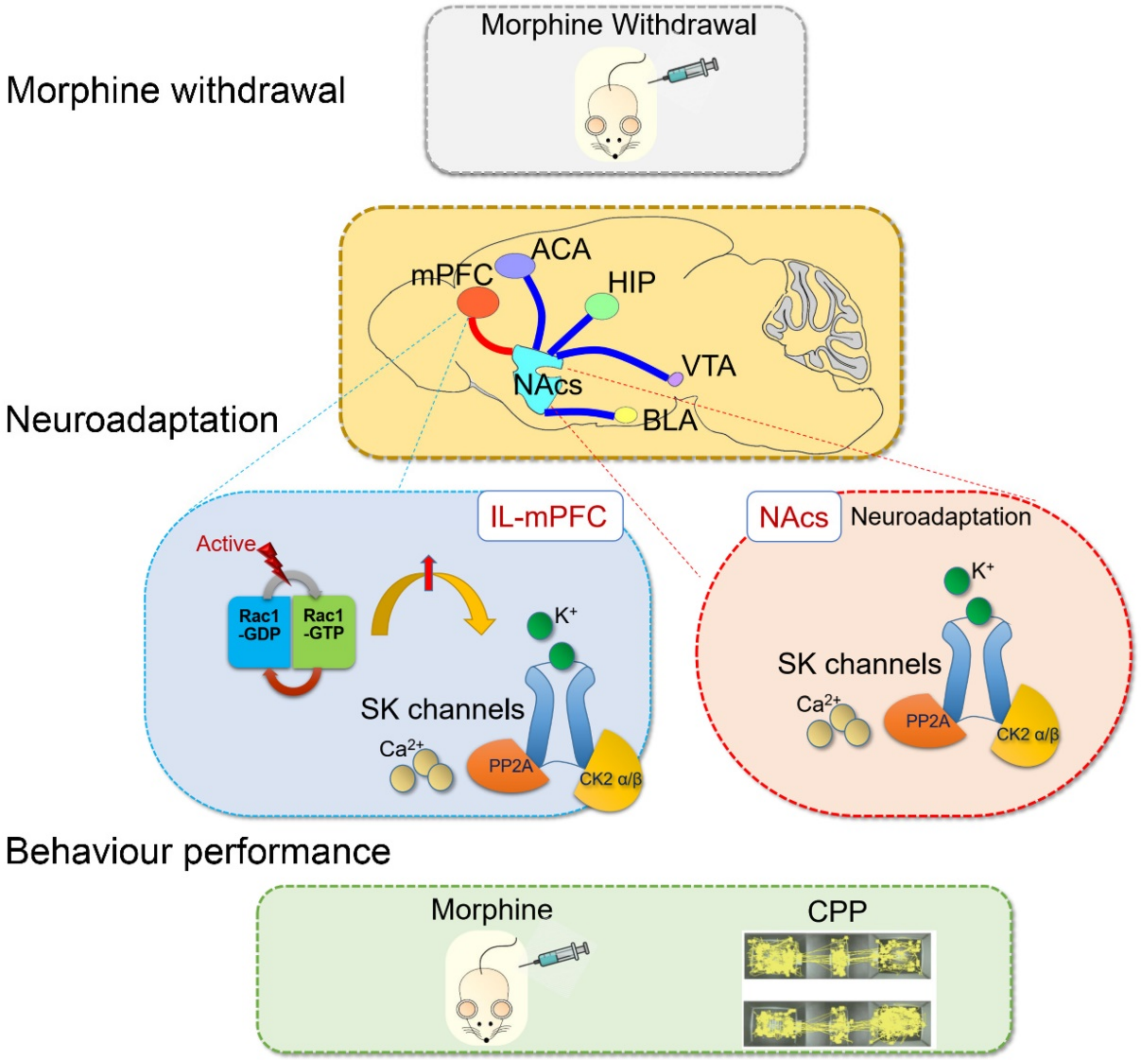
Summary: Rac1 in IL part of medial prefrontal cortex (IL-mPFC) differentially regulate the SK channel subtypes' expression and their activity to modulate morphine-induced neuroadaptations and CPP. ACA, anterior cingulate area; BLA, basolateral amygdala; HIP, hippocampus; mPFC, medial prefrontal cortex; VTA, ventral tegmental area; NAc, nucleus accumbens; CPP, conditioned place preference.

SK channels in the mPFC were upregulated by Rac1 signaling after one-week morphine withdrawal. SK channels and their upstream mechanisms play specific roles on neuroadaptations in the control-reward circuit during morphine withdrawal (summarized in **Figure 9).** Further studies are needed to determine the pathophysiological role of SK channels in the different stages of the addiction cycle 2 and their rate of recovery following more protracted drug withdrawal, which may throw light on the therapeutic mechanism of neuromodulation treatment for opioid dependence.

## Supplementary Material

Supplementary figures and tables.

## Figures and Tables

**Table 1:** Experiments performed, brain regions evaluated and sample sizes (number of rats)

Experiment	Regions	Control rats	MW rats
Electrophysiology	NAc (core & shell) and mPFC (IL & PrL)	22	24
Retrograde Tracers	NAc-mPFC	11	
Apamin related SK Currents	IL	8	9
SK2/SK3 expression	NAc and mPFC	12	12
PP2A and CK2 expression	NAc and mPFC	12	12
Activity of PP2A (Colorimetric Assay)	NAc and mPFC	8	8
LC-MS/MS	mPFC	12	12
Activity of Rac1	mPFC	6	6
Rac1 inhibition NSC23766	IL	9	
Rac1 genetic downregulation (CPP test & SK2 and SK3 expression)	IL	14	16

**Table 2:** The effect of downregulating Rac1 on the behavioral sensitization of locomotor activity during morphine exposure and withdrawal

Groups	SC rats with control shRNA (n=8)	SC rats with Rac1 shRNA (n=8)	MW rats with control shRNA (n=8)	MW rats with Rac1 shRNA (n=8)
Time point				
Day 0 (baseline)	3081.4 ± 306.7	3058.2 ± 286.3	3016.9 ± 321.2	3042.7 ± 276.6
Day 2 (drug exposure, 1^st^ day)	2954.0 ± 423.2	3042.2 ± 351.2	2714.3 ± 271.1	2852.3 ± 366.4
Day 8 (drug exposure, 7^th^ day)	3024.3 ± 356.2	3148.2 ± 375.0	2331.3 ± 221.0 *	2642.7 ± 376.3
Day 15 (drug withdrawal,7^th^ day)	2913.6 ± 357.7	2963.1 ± 386.3	4745.2 ± 521.2 *	3348.6 ± 362.5 ^#^

Data represent the locomotor activity of different groups of rats (45 min). Data correspond to means ± S.E.M., unit (cm), *P < 0.05 compared with SC rats with control shRNA. **^#^**P < 0.05 compared with MW rats with control shRNA.

## Abbreviations

AHP: action potential afterhyperpolarization
AP: action potential
DA: dopamine
DStr: dorsal striatum
CaM: calmodulin
IL: infralimbic
I/O slope: input/output slope
mAHP: medium AHP
mPFC: medial prefrontal cortex
MSNs: median spiny neurons
MW: morphine withdrawal
NAc: nucleus accumbens
NAcc: NAc core
NAcs: NAc shell
PFC: prefrontal cortex
PrL: prelimbic
SC: saline mock-treatment control
SK: small conductance calcium-activated potassium
TMR: tetramethylrhodramine-dextran
VTA: ventral tegmental area
