# Supplementary material for: Decreased Neuronal Excitability in Medial Prefrontal Cortex during Morphine Withdrawal is associated with enhanced SK channel activity and upregulation of small GTPase Rac1
Source: Theranostics. 2020 Jun 5;10(16):7369–83. doi: 10.7150/thno.44893 (PMC7330845; doi:10.7150/thno.44893)
Supplement: Supplementary file 1 — Supplementary figures and tables. [file thnov10p7369s1.pdf]

Decreased neuronal excitability in medial prefrontal cortex during morphine withdrawal is associated with enhanced SK channel activity and upregulation of small GTPase Rac1

Supplemental Information

Supplementary Methods and Materials
Supplementary Figures S1-S3
Supplementary Table S1-S3
Supplementary References

Supplementary Methods and Materials

Animals

Male Sprague Dawley rats (200–250 g) were acquired from the Animal Care Committee of the Fourth Military Medical University (FMMU). All procedures were conducted in compliant with the guidelines of the National Institutes of Health and the FMMU Animal Care and Use Committee, and this study was approved by the institutional ethical committee of Tangdu hospital, FMMU. All rats were housed in conventional open top cages with food and water available ad libitum under 12 hours light/dark cycle (lights on 8:00 a.m.) and constant temperature 22 ± 1°C and humidity 55±10%. All behavioral studies were performed between 9:00 a.m -11:00 a.m, and following every test, the apparatus was thoroughly cleaned with 20% v/v ethanol and dried to remove odor cues. Before initiating the conditioned place preference (CPP) pre-test animals were allowed to accustom to the laboratory conditions for 7 days.

Drugs

Morphine was acquired from Shenyang No. 1 Medical Drugs Company (Shenyang, China) and saline was obtained from Disai Biological Pharmaceutical Company (Xi'an, China). Saline (0.9%) or morphine (10 mg·kg^{-1}) administrations were injected subcutaneously for 7 consecutive days followed by one-week withdrawal.

Conditioned place preference procedure

All behavioral experiments were performed at 9 a.m -11 a.m during the light phase of the cycle. Methods for CPP were adapted from published procedures [1]. Rats were trained in the standard device (Noldus Information Technology Co., Ltd, Netherlands) consisting of two equally sized chambers and a smaller center chamber. The Behavior

was also recorded through a roof-camera using ETHOVISION 3.1 software (Noldus Information Technology Co., Ltd, Netherlands). The CPP score represents the time in the morphine treatment-paired compartment during the testing phase minus that during the preconditioning phase.

Brain slice preparation and ex-vivo electrophysiology
The patch clamping method used was based on a previous protocol, with certain modifications [1, 2]. Rats from saline mock-treatment control (SC) groups or morphine withdrawal groups were sedated by intraperitoneal (i.p.) injection with pentobarbital sodium (40 mg·kg^{-1}, Cat.# P11011, Merck, Germany) and then decapitated. Brains were quickly separated and immersed in ice-cold (0–3°C) slicing solution, which contains (in mM): 225 Sucrose; 119 NaCl, 2.5 KCl, 4.9 $MgCl_2$, 0.1 $CaCl_2$, 26.2 $NaHCO_3$, 1.0 NaH_2PO_4, 1.25 glucose; 1 ascorbic acid; and 3 kynurenic acid. Coronal slices (250-300 μm) containing the NAc or mPFC were cut in the same solution [3]. The slices were recovered at 32℃ in carbogen-bubbled (95% O_2, 5% CO_2) ACSF, containing (in mM): 126 NaCl, 2.5 KCl, 1.2 $MgCl_2$, 2.4 $CaCl_2$, 18 $NaHCO_3$, 1.2 NaH_2PO_4, 11 glucose (pH 7.2-7.4, 301-305 mOsm). During our experiments, the slices were immersed and continuously perfused with warmed carbogen-bubbled ACSF (32°C), while picrotoxin (50 μM; Sigma, USA) and CNQX (10 μM; Sigma, USA) were applied to block GABA-receptors and AMPA-type glutamate receptors. Our trials were focused to the GABAergic median spiny projection neurons, which accounts more than 90% of the efferent neurons within the NAc core and shell, while other cells could be separated easily by features of a large soma or very high firing rates and larger AHPs [4-6].

Whole-cell recordings were achieved for mPFC pyramidal neurons and NAc MSNs with the guidance of differential interference contrast microscopy (BX51WI; Olympus, Japan) and a CCD camera (Olympus, Japan). Borosilicate glass micropipettes (3-5 MΩ) were prepared by a P-97 horizontal micropipette puller (Axon Instr., USA). The intracellular solution used in whole-cell voltage and current clamp recordings contained (in mM): 130 KOH, 2.8 NaCl, 17 HCl, 20 HEPES, 105 methane sulfonic acid, 0.3 EGTA, 2.5 MgATP, 0.25 GTP (pH 7.2-7.4, 275-285 mOsm). EGTA was incorporated in the pipette solution to maintain calcium-dependent potassium currents during recordings [2]. To measure firing, we applied current pulses by a patch amplifier in current clamp mode, and applied a sequence of 7-8 current pulses (300 ms duration, 20 pA apart) for every 30 seconds. The minimum current amplitude was attuned for each neuron, in order to make the first pulse just under the spike firing threshold. The resting membrane potential was set to –90 mV before analysis of firing. For SK current measurement, neurons were held at –70 mV, then depolarized for 400 ms to steps ranging from –40 to –10 mV (with 10 mV between steps) prior to being brought back to –70 mV. The SK tail current was observed upon returning to –70 mV. Depolarizing pulses were combined with a 33.3 pA hyperpolarizing pulse to test the input resistance. We utilized the anterior commissure, the lateral ventricles and the dorsal striatum as landmarks for locating the position of the NAc shell, NAc core,

prelimbic cortex and infralimbic cortex for the patch clamping. The shape of NAc shell is ring-like in coronal section. The distance between NAc shell to AC is about 4 mm-13 mm. The NAc shell or layer 5 pyramidal cells located in the infralimbic subregion of the mPFC were visually recognized by using an upright infrared differential interference contrast microscope (BX51WI; Olympus, Japan).

Primary neuronal cultures
E18 pregnant Sprague-Dawley female rats were prepared for primary cortical cultures as previously described [7]. Briefly, embryos were obtained from the rats and then placed into Hank's Balanced Salt Solution (HBSS) (Cat. #14025134, Invitrogen, USA)-HEPES (10 mM; Cat.# H4034, Sigma, USA) solution. Prefrontal cortex was dissected from the embryos and incubated in 0.25% trypsin (Cat. #15400054, Invitrogen, USA) for 25 min at 37 °C with tapping every 5 min. Prefrontal cortex were washed for three times with HBSS-HEPES, and triturated with a fire-polished Pasteur pipette. Tissues were dissociated after 10-20 trituration, and neurons were immediately plated in pre-equilibrated dishes or plates coated with 1 $mg \cdot ml^{-1}$ poly-l-lysine (P2636, Sigma, USA) in plating medium. The plating medium consisted of Neurobasal medium (Cat. #21103049, Gibco, USA) supplemented with 2% FBS, 2% B-27 (Cat. #17504044, Gibco, USA), 2% glutamax (Cat. # 35050-061, Gibco, USA), and 2% penicillin–streptomycin (Cat. #15140122, Gibco, USA). The neurons were incubated at 37 °C in a humidified 5% CO_2 atmosphere, and primary culture medium was changed every 3 days or 4 days.

Brain stereotaxic injection of TMR into NAc for retrograde tract-tracing
All surgical procedures for 11 rats were deeply anesthetized with pentobarbital sodium (i.p., 40 $mg \cdot kg^{-1}$, Cat. # P11011, Merck, Germany). The anesthetized rats were placed onto a stereotaxic frame (NARISHIGE, Tokyo, Japan). According to the stereotaxic coordinates in the stereotaxic atlas of Paxinos and Watson (2007), 0.05 μl of 10% tetramethylrhodamine-dextran (TMR, D3308, 3,000 MW, Molecular Probe, Eugene, OR) dissolved in trisodium citrate solution (pH 3.0) was made stereotaxically into the bilateral NAc core or NAc shell of the rats (NAc core: 1.8 mm anterior to the Bregma, ± 1.4 mm to the midline, and 7.2 mm deep from the brain surface; NAc shell: 1.8 mm posterior to the Bregma, ± 0.8 mm to the midline, and 8.0 mm deep from the brain surface). A glass micropipette (internal tip diameter 15–25 μm) attached to a 1 μl Hamilton microsyringe was used. Each injection was made by pressure over a period of 10 min and the micropipette was left in the place for additional 20 min after the injection.

Immunohistochemistry
The animals were deeply sedated by intraperitoneal injection (40 $mg \cdot kg^{-1}$) of pentobarbital sodium (Cat.# P11011, Merck, Germany) and transcardially perfused with 100 ml of PBS and 4 % paraformaldehyde (PFA; Sigma-Aldrich, St. Louis, USA). Brain tissues were separated and post-fixed overnight with 4 % PFA at 4 °C, then cut into sections (30 μm) using a vibratome (VT1000S; Leica, Wetzlar,

Germany). Sections were washed in 0.1 M phosphate buffer prepared for TMR and NeuN immunostaining. The slices were incubated in PBS with 0.2% Triton X-100 (10 min), washed with PBS (3×5 min), blocked in 1% normal horse serum in 0.1 M phosphate buffer (30 min, room temperature), and subsequently incubated overnight at 4°C with the following primary antibodies: mouse monoclonal anti-NeuN (Cat.# MAB377, 1:1000; Millipore, Billerica, United States), rabbit anti-TMR monoclonal antibody (Cat.# A-6397, 1:400, Invitrogen, United States) in PBS. Following washing in PBS (3×5 min), Cy2-conjugated anti-mouse IgG (Cat.# 115-225-071, 1:200, Jackson ImmunoResearch Laboratories, United States) and Cy3-conjugated anti-rabbit IgG (Cat.# 111-095-003, 1:200, Jackson ImmunoResearch Laboratories, United States) were used for fluorescence detection. Nuclei counterstaining was performed using 4',6-Diamidino-2-phenylindole dihydrochloride (DAPI; Cat.# D9542, Sigma-Aldrich, United States). Fluorescence images were taken using a confocal microscope (A1; Nikon, Japan).

Western blotting

The SK2 and SK3 subunits protein expression level was examined in the mPFC, NAc and dorsal striatum during drug withdrawal as described previously [1, 8]. Lysis buffer

(50 mM Tris-HCl, 150 mM NaCl, 1% Triton X-100, 0.5% sodium deoxycholate, 0.1% sodium dodecyl sulfate, pH 8.0), supplemented with 1% protease inhibitor cocktail (P8340; Sigma Aldrich, USA). The following antibodies were used: Anti-SK2 C-terminus (Cat.# APC-045, 1:800, Alomone, Jerusalem, Israel), Anti-SK3 N-terminus (Cat.# APC-025, 1:800, Alomone, Jerusalem, Israel), Anti-Rac1(Cat.# ab33186, 1:200, Abcam, United States), Anti-PP2A (Cat.# SAB4502298, 1:1000, Sigma-Aldrich, United States), Anti-CK2α (Cat.# SAB4500514, 1:800, Sigma-Aldrich, United States), Anti-CK2β (Cat.# SAB4500516, 1:800, Sigma-Aldrich, United States), Anti-β-actin (1:5000, TA-09; ZSGB-BIO Co., Beijing, China) and Anti-β-tubulin (Cat.# SAB4500088, 1:1000, Sigma-Aldrich, United States). After lysing fresh samples in lysis buffer, the protein concentration was determined using a bicinchoninic assay kit (Beyotime, Ltd., Haimen, China) according to the kit manufacturer's protocol. Equal quantities of protein from the NAc, dorsal striatum or mPFC were resolved on 8% acrylamide SDS-PAGE gels and electrophoretically transferred to PVDF membranes (Millipore, Billerica, MA, USA). The membranes were blocked for 2 hours in 5% skim milk diluted in PBS/tween (PBST, 0.01 M PBS with 0.1% Tween 20) at 37 °C with gentle shaking. The membranes were then incubated overnight with antibodies reactive against the primary antibodies (overnight at 4 °C in 4% skim milk). Then, the membranes were incubated with the HRP-conjugated secondary antibodies after PBST washing. Blots were developed with chemiluminescence (Chemi Doc XRS Plus; Bio-Rad, CA, USA). ImageJ 4.0 (National Institutes of Health, Bethesda, MD, USA) was applied to quantify and analysis the band intensity. The expression of SK2, SK3 and Rac1 was normalized to that of β-actin, the expression of PP2A, CK2α and CK2β was normalized to that of β-tubulin.

PP2A activity assay

PP2A activity was measured as previously reported using the PP2A Colorimetric Assay kit (GenMed Scientifics, Woodland, CA, USA) [9]. This assay is based on the release of free phosphate from the dephosphorylation of RKpTIRR by endogenous PP2A, which is detected via chromogenic reaction with molybdenum blue produced by ferrous sulfate reduction. The free phosphate concentration was measured at 660 nm on a spectrophotometer (Bio-Rad). The phosphate concentration (μM/L) was converted to PP2A activity/mg protein as described by the manufacturer.

LC-MS/MS iTRAQ analysis

The methods of sample preparation for LC-MS/MS iTRAQ analysis were reported in previous study [10, 11]. For the iTRAQ analysis, in each group, fresh mPFC tissues were rapidly dissected from the brains and sampled. To reduce individual variation, 12 SC rats were pooled into 3 samples as saline1, saline2, and saline3, and 12 MW rats were pooled into 3 samples as M1, M2 and M3. The pooled samples were digested according to the FASP procedure and labeled using the 8-plex iTRAQ reagent according to the manufacturer's instructions (Applied Biosystems). The final proteins that were deemed to be differentially expressed were filtered as a p value <0.05 and 1.1-fold changes (>1.10 or <0.91) relative to the SC group.

Functional classification and Gene Ontology (GO) enrichment analyses of the DEPs were carried out using DAVID (https://david.ncifcrf.gov/). Proteins were classified by GO category (http://www.geneontology.org), including "biological process," "cell component," and "molecular function." The KEGG (http://www.genome.jp/kegg/) database was employed to identify significantly enriched pathways. Rattus norvegicus was selected as the species and the background. The significance was determined with slight modifications as recommended by the authors of DAVID according to the Benjamini-corrected P value <0.05. Functional protein association networks were explored in STRING v.10.5 (http://string-db.org/).

A total of 131 differentially expressed proteins were annotated by GO analysis and were classified into 24 significant GO terms in Biological Process (**Supplementary Figure S2A**), 17 in Cellular Component (**Supplementary Figure S2B**), and nine in Molecular Function (**Supplementary Figure S2C**). Notably, these proteins were found to be enriched in GO terms which associated with potassium channel activity and regulation of Cytoskeletal component (**Supplementary Figure S2D**).

Assay for Rac1 activity

Active Rac1 pull-downs were performed following the active Rac1 Pull-Down and Detection Kit (catalog #16118, Thermo Scientific™) protocol [12]. Briefly, lysates of the rat mPFC tissue was centrifuged (16,000 × g at 4°C for 15 min), and then the transferred supernatants were added with GTPγS or GDP to incubate at 30°C for 15 min. The mixtures were incubated with glutathione resin beads and glutathione

S-transferase-fused Rac1-binding domain of p21-activated kinase (Pak) at 4°C for 1 h. The beads and proteins bound to the fusion protein were washed at 4°C, eluted in SDS sample buffer, and analyzed for bound Rac1 by Western blotting.

lentivirus construction

In vivo experiments, lentiviruses were generated from Genepharma Technology Co., Ltd (Shanghai, China). Lentivirus plasmid pSicoR was purchased from Addgene, and oligos coding for the various shRNAs were annealed and cloned into HpaI-XhoI-digested pSicoR vectors. The target shRNA regions were chosen as follows: Rac1-124, GCCAATGTTATGGTAGATGGA; Rac1-219, GCAAACAGACGTGTTCTTAAT; Rac1-340, GGGACGAAGCTTGATCTTAGG; negative control, TTCTCCGAACGTGTCACGT.

Stereotaxic injections of lentivirus into the IL cortex

Animals were deeply anesthetized with an i.p. injection (40 $mg \cdot kg^{-1}$) of pentobarbital sodium (Aoxin Chemical Factory, Yangzhou, China). Lentiviruses (9×10^8 to 1×10^9 TU/ml) were stereotaxically injected into the IL (2.5 L/site) over 5 min using a glass micropipette (internal tip diameter 15-25 μm) attached to a 5 μl Hamilton microsyringe. The injector was retained in place for another 10 min then withdrawn at 1 mm/min. We applied the injections bilaterally at the following coordinates (as calculated from bregma and the dura mater): 3.2 mm posterior to the Bregma, ± 0.6 mm to the midline, and 5.0 mm deep from the brain surface.

Adeno-associated virus (AAV) construction and infection

In vitro experiments, cortical neurons were transfected with AAV-mediated gene delivery as described in [13]. The transductions were performed at 7 DIV and maintained for 12 DIV. The target shRNA regions were chosen as follows: Rac1-124, GCCAATGTTATGGTAGATGGA; Rac1-219, GCAAACAGACGTGTTCTTAAT; Rac1-340, GGGACGAAGCTTGATCTTAGG; negative control, TTCTCCGAACGTGTCACGT. For the rescue plasmid, the shRNA targeting binding sites of rac1 plasmid were synonymously mutated to prevent the above three shRNAs from interfering with the expression of Rac1. The synonymous mutation sites of rac1 plasmid are as follows: site-124, GCCAATGTAATGGTCGACGGT; site-219, GCAAACAGATGTATTTTTGAT; site-340, GGGACGAAGCTAGACCTGAGA. The AAV-Rac1 shRNA, AAV-Rac1 rescue and AAV-Ctrl-shRNA of AAV2/9 serotype were packaged by Genepharma Technology Co., Ltd (Shanghai, China). shRNA plasmid pSicoR was purchased from Addgene, and oligos coding for the various shRNAs were annealed and cloned into HpaI-XhoI-digested pSicoR vectors. Viral titers over 1×10^{12} genomic particles/mL were used. The cells were transduced with AAVs at a multiplicity of infection (MOI) of 10^4 viral genome copies per cell (VGC/cell) for 3 h at 37°C. The media were subsequently completed with B27-supplemented Neurobasal medium.

The assessment of locomotor sensitization

The locomotor activity of each rat was measured 45 min using locomotor activity cage (Noldus Information Technology Co., Ltd, Netherlands) as described previously [14]. Morphine withdrawal responses (such as wet dog shakes) and locomotor activity were simultaneously observed for the same duration. Other withdrawal symptoms (such as the number of fecal pellets, ptosis and diarrhea) were not included here.

Statistical analysis

pClamp 10.2 (Axon Instr., USA) and Origin 9.0 (Origin Lab, Northampton, MA, USA) were applied for analyzing results of ex-vivo electrophysiological recordings. Considering varied number of neurons recorded for each rat, we averaged the baseline spike firing and voltage clamp parameters (baseline input/output slope, action potential and input resistance parameters, tail currents, etc.) for all neurons achieved from a given animal, and acquired a specific value of each of these parameters for each individual rat. The data were expressed as means ± SEM for all the tests. All statistics were presented using an unpaired t-tests, otherwise noted. All tests were two-sided and the statistical significance was set at 0.05.

Supplementary Figures

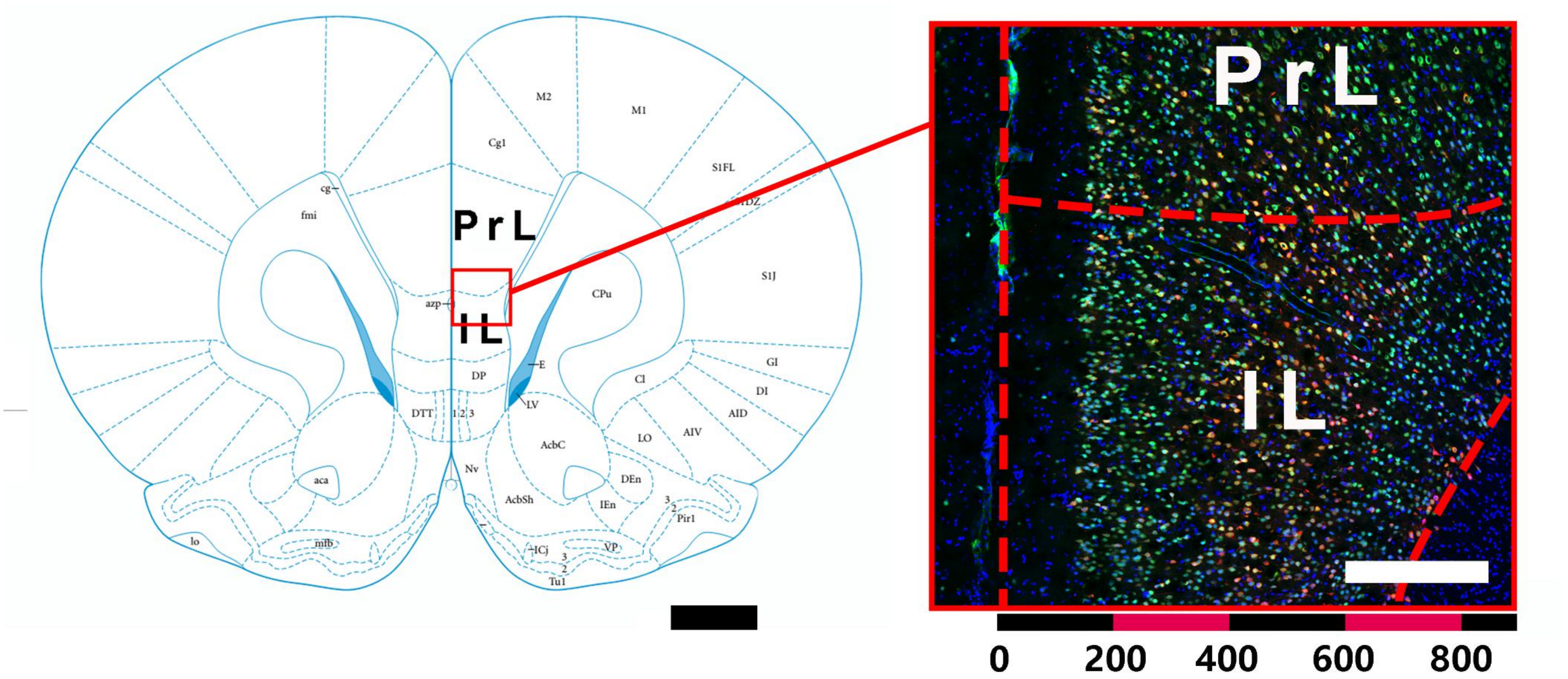

Figure S1. A coronal section of retrogradely labeled neurons in mPFC from TMR injections in NAc shell. n=11, scale bar 1 mm in main panel, 250 μm in inset.

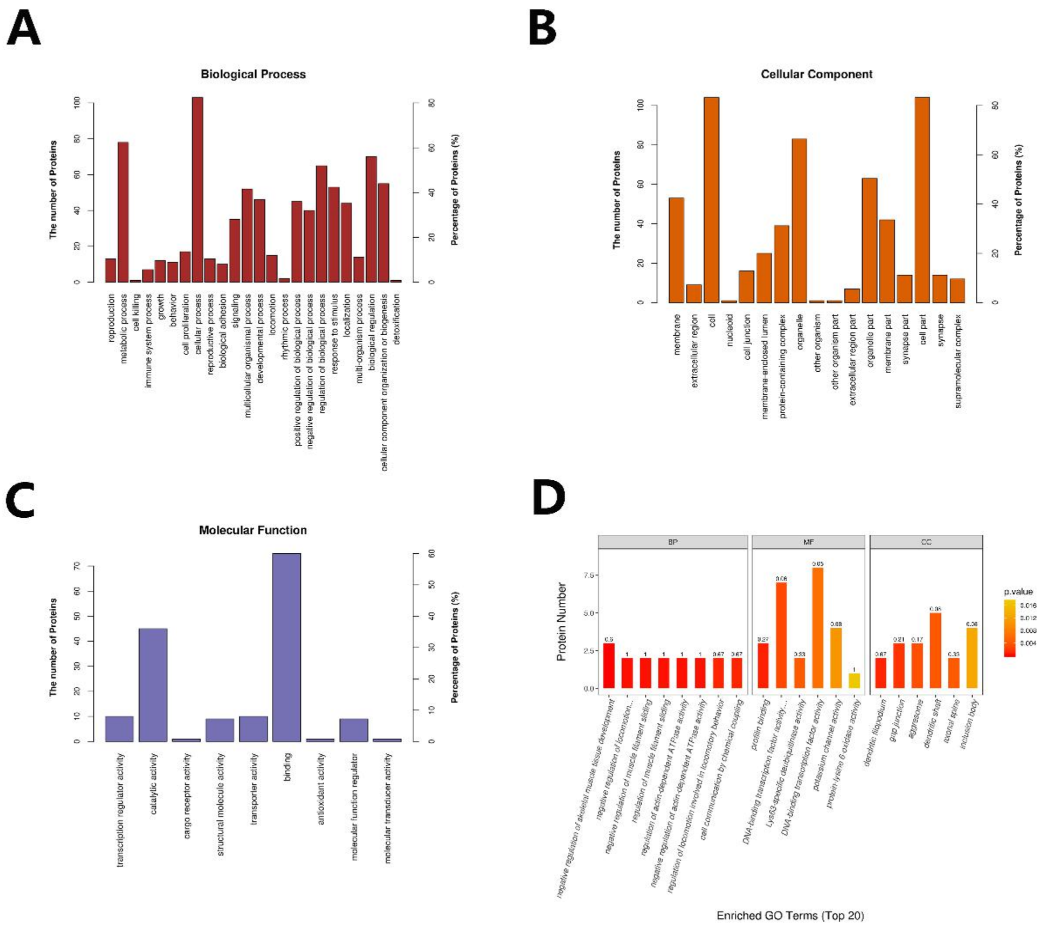

Figure S2. GO annotation and functional classification of DEPs: annotated terms for biological process (**A**), molecular functions (**B**), cellular component (**C**) and enriched GO Terms (**D**).

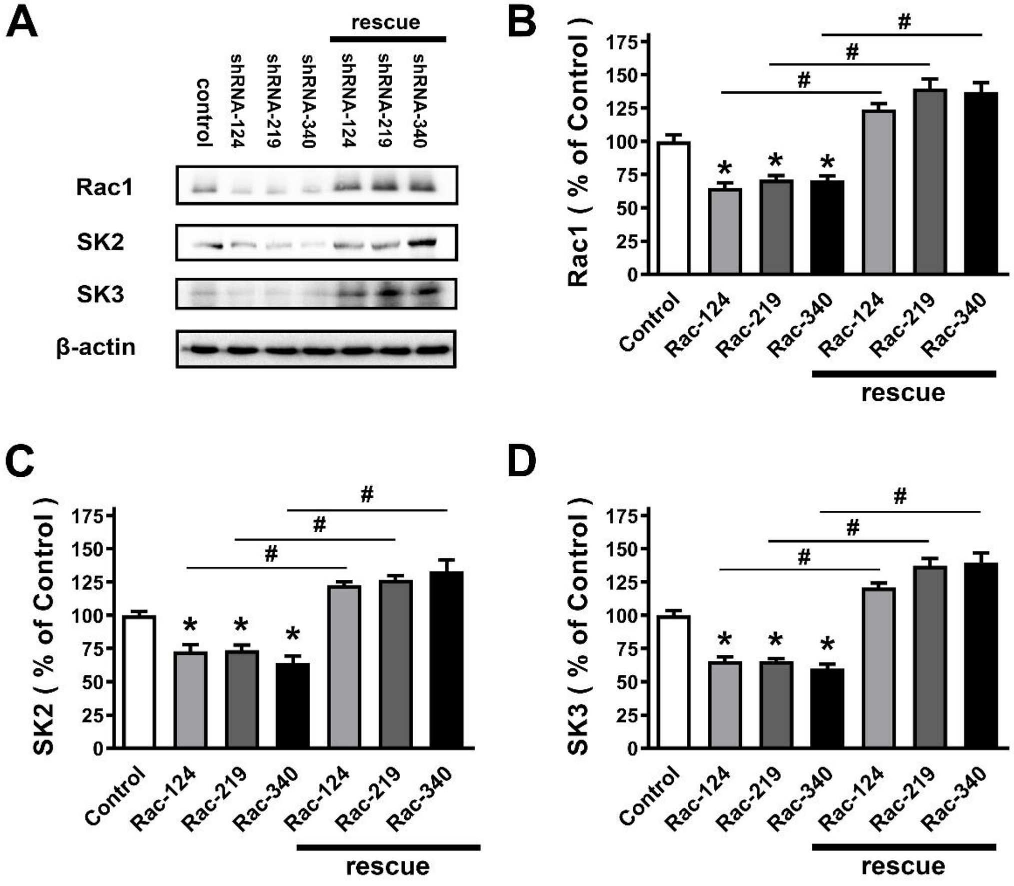

Figure S3. Genetically manipulating Rac1 regulates protein expression levels of SK2 and SK3 subtype channels in primary cortical neurons. **A** Knockdown of the Rac1 expression with Rac1-shRNA suppressed protein expression level of SK2 and SK3, and then rescued by AAV expressing Rac1 in cortical neurons. **B-D** Quantitative analysis of Rac1, SK2 and SK3 in **A**, normalized to β-actin. Data correspond to means ±

S.E.M., n= 6, one-way ANOVA with Bonferroni's multiple comparison; Rac1: control 100.0 ± 4.96 %, shRNA Rac124 64.7 ± 3.98 %, shRNA Rac219 71.2 ± 3.18%, shRNA Rac340 70.5 ± 3.35%, shRNA Rac124 + rescue 124.0 ± 4.42 %, shRNA Rac219 + rescue 139.8 ± 7.31 %, shRNA Rac340 + rescue 137.2 ± 6.82 %; SK2: control 100.0 ± 2.88 %, shRNA Rac124 72.9 ± 5.04 %, shRNA Rac219 73.7 ± 3.94 %, shRNA Rac340 64.25 ± 5.01 %, shRNA Rac124 + rescue 122.7 ± 2.59 %, shRNA Rac219 + rescue 126.8 ± 3.03 %, shRNA Rac340 + rescue 133.2 ± 8.47 %; SK3: control 100.0 ± 3.67 %, shRNA Rac124 65.3 ± 3.40 %, shRNA Rac219 65.5 ± 1.92 %, shRNA Rac340 60.0 ± 3.19 %, shRNA Rac124 + rescue 120.9 ± 3.35 %, shRNA Rac219 + rescue 137.3 ± 5.46 %, shRNA Rac340 + rescue 139.9 ± 7.06 %; *P < 0.05 vs. control shRNA, $^{\#}$P < 0.05 rescue groups vs. shRNA-Rac1 groups.

Table S1. All statistical analyses according to figures in the text

Figure	Response variable	groups	n define as	Statistical methods	Degrees of freedom and F/t/p value	Post hoc test	Significance
1B	Changed time on drug-paired side	saline n=68<br>morphine n=71	rat	unpaired t-test	t=3.790, df=137		p=0.0007
1D	Frequency of AP	NAcc-180pA-saline n=8<br>NAcc-180pA-morphine n=8<br>NAcc-220pA-saline n=8<br>NAcc-220pA-morphine n=8	rat/ 14 neurons	Two-way ANOVA	treatment, $F_{(1, 28)}$=0.0337, p=0.8556; applied current, $F_{(1, 28)}$=32.81, p<0.0001; treatment*applied current, $F_{(1, 28)}$=0.3096, p=0.5823	Bonferroni's multiple comparison	NAcc-180pA-saline vs. NAcc-180pA-morphine, p=0.8439 NAcc-220pA-saline vs. NAcc-220pA-morphine, p=0.9576
1F	Frequency of AP	NAcs-180pA-saline n=8<br>Nacs-180pA-morphine n=8<br>Nacs-220pA-saline n=8	rat/ 12 neurons	Two-way ANOVA	treatment, $F_{(1, 28)}$=17.05, p=0.0003; applied current, $F_{(1, 28)}$=58.86, p<0.0001; treatment*applied current, $F_{(1, 28)}$=1.893, p=0.1797	Bonferroni's multiple comparison	NAcs-180pA-saline vs. NAcs-180pA-morphine, p=0.045; NAcs-220pA-saline vs. NAcs-220pA-morphine, p=0.003

		Nacs-220pA-morphine<br>n=8					
**Figure**	**Response variable**	**groups**	**n define as**	**Statistical methods**	**Degrees of freedom and F/t/p value**	**Post hoc test**	**Significance**
2D	Density of double labeled neurons	NAcc-PrL n = 15<br>NAcc-IL n = 15<br>NAcs-PrL n = 18<br>NAcs-IL n = 18	slice / 11 rats	One-way ANOVA	F=9.452, df=62	Bonferroni's multiple comparison	NAcs-PrL vs. NAcs-IL,p= 0.0[illegible]<br>NAcc-IL vs. NAcs-IL,p=0.00[illegible]<br>NAcc-PrL vs. NAcs-IL,p=0.0[illegible]
2F"	I/O slope	SW n = 14<br>MW n = 16	rat/ 46 neurons	unpaired t-test	t = 0.226, df=28		p = 0.823
3B	Peak tail current	Saline-baseline<br>n=9<br>Morphine-baseline<br>n=8<br>Saline-after apamin<br>n=9<br>Morphine-after apamin<br>n=8	rat/ 14 neurons	Two-way RM-ANOVA	apamin: $F_{(1,30)}$ = 14.20, p = 0.0196; group: $F_{(1,30)}$ = 640.8, p < 0.001; apamin x group: $F_{(1,30)}$ = 15.54, p = 0.0169	Bonferroni's multiple comparison	Saline-baseline vs. Morphine-baseline,p=0.0186
3C	Peak tail current	Saline--40mV n=9<br>Morphine--40mV n=8<br>Saline--30mV n=9	rat/ 23 neurons	Two-way RM-ANOVA	voltage: $F_{(3,60)}$ = 232.87, p < 0.001; group: $F_{(3,60)}$ = 4.126, p < 0.001; voltage	Bonferroni's multiple comparison	Saline--20mV vs. Morphine--20mV,p=0.0216;Saline[illegible] vs. Morphine--10mV,p=0.0134

		Morphine--30mV n=8<br>Saline--20mV n=9<br>Morphine--20mV n=8<br>Saline--10mV n=9<br>Morphine--10mV n=8			x group: $F_{(9,60)} = 3.164$, $p < 0.001$		
**Figure**	**Response variable**	**groups**	**n define as**	**Statistical methods**	**Degrees of freedom and F/t/p value**	**Post hoc test**	**Significance**
4B left panel	Relative protein level of SK2 (normalized to β-actin)	saline n=7<br>morphine n=7	rat	unpaired t-test	t = 0.1878, df=12		p = 0.856
4B right panel	Relative protein level of SK3 (normalized to β-actin)	saline n=8<br>morphine n=8	rat	unpaired t-test	t = 3.050, df=14		p = 0.033
4D left panel	Relative protein level of SK2 (normalized to β-actin)	saline n=6<br>morphine n=6	rat	unpaired t-test	t =3.056,, df=10		p = 0.022

Figure	Response variable	groups	n define as	Statistical methods	Degrees of freedom and F/t/p value	Post hoc test	Significance
4D right panel	Relative protein level of SK3 (normalized to β-actin)	saline n=6<br>morphine n=6	rat	unpaired t-test	t = 4.257, df=10		p = 0.005
4F left panel	Relative protein level of SK2 (normalized to β-actin)	saline n=8<br>morphine n=8	rat	unpaired t-test	t = 1.403, df=14		p = 0.210
4F right panel	Relative protein level of SK3 (normalized to β-actin)	saline n=8<br>morphine n=8	rat	unpaired t-test	t = 0.482 , df=14		p = 0.647
5B left panel	Relative protein level of PP2A (normalized to β-tubulin)	saline n=7<br>morphine n=7	rat	unpaired t-test	t = 0.414, df=12		p = 0.693

Figure	Response variable	groups	n define as	Statistical methods	Degrees of freedom and F/t/p value	Post hoc test	Significance
5B middle panel	Relative protein level of CK2α (normalized to β-tubulin)	saline n=7<br>morphine n=7	rat	unpaired t-test	t = 0.205 , df=12		p =0.843
5B right panel	Relative protein level of CK2β (normalized to β-tubulin)	saline n=7<br>morphine n=7	rat	unpaired t-test	t = 3.185 , df=12		p =0.019
5D left panel	Relative protein level of PP2A (normalized to β-tubulin)	saline n=8<br>morphine n=8	rat	unpaired t-test	t = 3.147, df=14		p =0.020
5D middle panel	Relative protein level of CK2α (normalized to β-tubulin)	saline n=8<br>morphine n=8	rat	unpaired t-test	t = 3.935 , df=14		p =0.008

Figure	Response variable	groups	n define as	Statistical methods	Degrees of freedom and F/t/p value	Post hoc test	Significance
5D right panel	Relative protein level of CK2β (normalized to β-tubulin)	saline n=8<br>morphine n=8	rat	unpaired t-test	t = 0.208 , df=14		p =0.842
5E	Relative PP2A Activity (normalized to saline group)	saline n=6<br>morphine n=6	rat	unpaired t-test	t = 2.975 , df=10		p =0.025
5F	Relative PP2A Activity (normalized to saline group)	saline n=6<br>morphine n=6	rat	unpaired t-test	t = 2.906 , df=10		p =0.027
6C upper left panel	Relative protein level of RAB2B (normalized to Reference)	saline n=3<br>morphine n=3	one randomised mix / 4 rats	unpaired t-test	t=4.189, df=4		p = 0.0138

Figure	Response variable	groups	n define as	Statistical methods	Degrees of freedom and F/t/p value	Post hoc test	Significance
6C upper middle panel	Relative protein level of RAB3B (normalized to Reference)	saline n=3<br>morphine n=3	one randomised mix / 4 rats	unpaired t-test	t=3.830, df=4		p = 0.0186
6C upper right panel	Relative protein level of RhoA (normalized to Reference)	saline n=3<br>morphine n=3	one randomised mix / 4 rats	unpaired t-test	t=10.55, df=4		p = 0.0005
6C bottom left panel	Relative protein level of RhoC (normalized to Reference)	saline n=3<br>morphine n=3	one randomised mix / 4 rats	unpaired t-test	t=6.105, df=4		p = 0.0036
6C bottom middle panel	Relative protein level of Rac1 (normalized to Reference)	saline n=3<br>morphine n=3	one randomised mix / 4 rats	unpaired t-test	t=9.525, df=4		p = 0.0007

Figure	Response variable	groups	n define as	Statistical methods	Degrees of freedom and F/t/p value	Post hoc test	Significance
6C bottom right panel	Relative protein level of RRas (normalized to Reference)	saline n=3<br>morphine n=3	one randomised mix / 4 rats	unpaired t-test	t=4.015, df=4		p = 0.0159
6E	Relative Rac1-GTP (normalized to Rac1)	saline n=10<br>morphine n=10	rat	unpaired t-test	t=7.008, df=18		p = 0.0004
7C	Peak tail current	Baseline n=9<br>After NSC23766 n=9<br>Wash n=9	rat / 13 neurons	One-way ANOVA	F=84.32, df=24	Bonferroni's multiple comparison	Baseline vs. After NSC23766, p<0.[illegible] Baseline vs. Wash,p= 0.3826; A[illegible] NSC23766 vs. Wash, p <0.000[illegible]
8C	Relative protein level of Rac1 (normalized to β-actin)	control shRNA n=8<br>Rac1 shRNA n=10	rat	unpaired t-test	t = 6.524, df=16		p =0.0006
**Figure**	**Response variable**	**groups**	**n define as**	**Statistical methods**	**Degrees of freedom and F/t/p value**	**Post hoc test**	**Significance**

8D	Conditioning score	control shRNA- pre-test n=8<br>Rac1 shRNA- pre-test n=8<br>control shRNA- test n=8<br>Rac1 shRNA- test n=8	rat	Two-way RM-ANOVA	main effect of gene: $F_{(1,28)} = 5.432$, $p = 0.0272$, test: $F_{(1,28)} = 7.118$, $p = 0.0125$; main effect of gene x test: $F_{(1,28)} = 3.988$, $p = 0.0556$	Bonferroni's multiple comparison	control shRNA- test vs. Rac1 sh[illegible] test, p=0.0236; control shRNA- [illegible] vs. control shRNA- test, p= 0.0[illegible]
8F left panel	Relative protein level of SK2 (normalized to β-actin)	control shRNA n=6<br>Rac1 shRNA n=6	rat	unpaired t-test	t = 2.867, df=10		p = 0.0323
8F right panel	Relative protein level of SK3 (normalized to β-actin)	control shRNA n=6<br>Rac1 shRNA n=6	rat	unpaired t-test	t = 2.485, df=10		p = 0.0168

Supplementary Table S2. Differential expression of total Small GTPases between SC group and morphine withdrawal group (tested by LC-MS/MS iTRAQ)

Protein	Accession	Unique Peptides	Peptides	PSMs	AAs	MW [kDa]	calc. pI	Saline-1/REF	Saline-2/REF	Saline-3/REF	average Saline	M-1/REF	M-2/REF	M-3/REF	average M	Saline/M	M/Saline	t test p value
RAB21	ENSRNOP00000005258	9	9	24	223	24.1482	7.94287	1.03319	0.99193	1.00801	1.01104	1.01517	0.97764	0.99651	0.99644	1.01465	0.98556	0.4176
RAB5A	ENSRNOP00000070031	1	3	9	61	6.54742	10.0083	0.74009	0.85883	1.2323	0.94374	1.03937	0.98704	1.09604	1.04081	0.90673	1.10286	0.5568
RAB5C	ENSRNOP00000031520	7	11	76	216	23.4108	8.41162	0.99895	1.02566	1.0075	1.01071	1.00702	1.0067	0.98503	0.99958	1.01113	0.989	0.3582
RAB5B	RAB5B, member RAS oncogene family	9	58	215	23.6599	8.1333	0.97513	1.01942	0.96738	0.98731	0.9914	0.99939	0.98318	1.00476	0.9958	0.99558	1.00444	0.8025
RAB22A	ENSRNOP00000061148	2	3	6	194	21.7611	8.14795	1.01781	0.98334	0.93656	0.97923	1.00681	1.01944	1.00751	1.01125	0.96834	1.0327	0.2514
RAB13	ENSRNOP00000071741	1	4	37	203	22.8869	9.43701	1.03168	0.96676	1.04881	1.01575	1.03431	0.99591	0.9636	0.99794	1.01785	0.98247	0.6105
RAB35	ENSRNOP00000029070	9	12	123	201	23.0108	8.29443	0.95487	1.03215	0.98495	0.99066	0.99388	1.0143	1.00887	1.00568	0.98506	1.01517	0.5542
RAB30	ENSRNOP00000068836	4	5	46	203	23.0435	4.97021	1.02808	0.98201	0.99222	1.00077	0.94391	0.97915	1.02896	0.98401	1.01703	0.98326	0.5863
RAB8B	ENSRNOP00000024287	4	12	103	207	23.5881	9.0708	1.00656	0.97521	1.01842	1.00006	1.02835	0.98621	1.00626	1.00694	0.99317	1.00688	0.7178

RAB6						23.57	5.541				1.005		0.9722	0.9903	0.992	1.0138	0.9863	0.77
A	ENSRNOP00000073670	1	15	110	208	49	5	0.93558	1.0842	0.99737	72	1.0135	1	3	01	1	8	47
RAB6						23.44	5.528				1.004	1.0106	0.9850	0.9715	0.989	1.0151	0.9850	0.77
B	ENSRNOP00000068600	6	14	119	208	69	81	0.94918	1.09678	0.96632	09	5	7	3	09	7	5	01
RAB8						23.65	9.070				1.005	1.0151	0.9821	0.9963	0.997	1.0071	0.9928	0.59
A	ENSRNOP00000020748	3	11	101	207	32	8	1.02054	0.99358	1.00095	02	5	7	2	88	6	9	80
RAB1						22.66	6.214				0.993	1.0011	1.0118	1.0105	1.007	0.9856	1.0145	0.27
A	ENSRNOP00000073493	8	16	195	205	34	36	0.99208	1.01309	0.97484	34	2	5	1	83	2	9	84
RAB1						22.96	5.236				0.995	0.9862	1.0143	1.0006	1.000	0.9946	1.0053	0.83
8	ENSRNOP00000025828	13	13	69	206	16	82	0.95148	1.02607	1.00767	07	9	5	3	42	5	8	35
RAB2						24.60	5.541				0.994	1.0137	0.9835	1.0111	1.002	0.9915	1.0085	0.76
7B	ENSRNOP00000016369	6	6	20	218	43	5	1.02202	1.01481	0.94621	35	5	4	4	81	6	1	13
RAB9						22.88	5.655				1.005	0.9732	0.9890	1.0415	1.001	1.0040	0.9959	0.86
A	ENSRNOP00000050986	3	4	8	201	12	76	1.01946	0.99861	0.99802	36	9	4	3	29	7	5	09
RAB1						24.47	5.935				0.992	1.0077	1.0080	0.9928	1.002	0.9898	1.0102	0.32
1B	ENSRNOP00000010197	13	13	85	218	35	06	0.9814	1.00697	0.98987	75	8	2	6	89	9	1	47
RAB2						22.79	6.536				0.977	1.0309	1.0029	0.9974	1.010	0.9672	1.0338	0.16
A	ENSRNOP00000008522	5	13	89	206	04	62	0.94509	1.00118	0.98597	41	7	5	9	47	9	2	86
RAB1						22.52	8.382				1.000	1.0094	1.0093	0.9926	1.003	0.9967	1.0032	0.75
0	ENSRNOP00000065234	14	19	127	200	66	32	1.01178	1.00527	0.98482	63	9	9	9	86	8	3	95
RAB1						27.36	7.972				1.011		1.0025	1.0123	0.998	1.0137	0.9864	0.55
2	ENSRNOP00000059602	4	10	68	245	6	17	1.04987	0.99454	0.99117	86	0.9796	3	4	16	3	6	62
RAB3						24.76					0.957	1.2144	1.1324	1.0707	1.139	0.8408	1.1892	0.01
B	ENSRNOP00000010645	7	12	113	219	91	5.021	0.93143	0.93954	1.00274	9	7	5	2	21	5	8	86
RAB2						25.05	5.249				0.988	1.0010	0.9627	1.0931	1.018		1.0312	0.50
7A	ENSRNOP00000068946	1	1	1	221	23	51	1.0186	0.96664	0.97901	08	1	6	1	96	0.9697	5	05

RAB3						25.85	5.236				0.979	1.0469	1.0154	1.0149	1.025	0.9552	1.0468	0.15
C	ENSRNOP00000015871	5	13	132	227	56	82	0.99668	1.01021	0.93277	89	4	8	3	78	6	4	37
RAB3						25.73	7.693				1.014	1.0011	0.9945	1.0047	1.000	1.0142	0.9859	0.26
3B	ENSRNOP00000017396	4	9	60	229	58	85	1.01934	1.0296	0.99418	37	2	3	6	14	4	6	29
RAB1						24.26	5.516				1.001	0.9792	1.0266	0.9964	1.000	1.0003	0.9996	0.98
5	ENSRNOP00000010043	9	11	81	212	81	11	0.99448	0.99733	1.01149	1	5	2	7	78	2	8	37
RAB3						24.95	5.033				0.992	0.9871	1.0426	0.9857	1.005	0.9875	1.0126	0.60
A	ENSRNOP00000026392	6	15	256	220	41	69	0.97038	1.01167	0.99599	68	7	6	8	2	4	2	38
RAB2							6.785				0.963	1.0186	1.0212	1.0308	1.123		1.1656	0.00
B	ENSRNOP00000057211	4	12	71	215	24.07	64	0.94138	0.96149	0.98884	9	6	1	3	57	0.8579	4	99
RAP2						20.62	4.817				0.974	1.0192	1.0256	1.0099	1.018	0.9574	1.0444	0.26
A	ENSRNOP00000068867	5	9	58	183	93	87	0.92516	1.0367	0.96302	96	9	1	5	28	6	3	03
RAB2						20.62	4.817				0.974	1.0192	1.0256	1.0099	1.018	0.9574	1.0444	0.26
6	ENSRNOP00000068867	5	9	58	183	93	87	0.92516	1.0367	0.96302	96	9	1	5	28	6	3	03
RAB3						24.27	4.919				1.009	0.9115	0.9997	1.0421	0.984	1.0252	0.9753	0.73
D	ENSRNOP00000015609	1	10	111	219	48	43	0.9455	1.12085	0.96164	33	3	8	8	5	3	9	31
RAB1						23.88	6.214				1.003	1.0012	1.0033	0.9885	0.997	1.0057	0.9943	0.63
4	ENSRNOP00000025649	11	13	111	215	19	36	0.98436	1.01838	1.00756	44	7	5	2	71	4		19
						23.24	6.049				0.992	0.9917	1.0131		0.993	0.9986	1.0013	0.96
RALB	ENSRNOP00000003413	6	9	40	205	58	32	0.95573	0.98411	1.0374	41	8	8	0.9764	79	2	8	07
RAB7						23.48	6.697				0.987	1.0175	1.0002	1.0019	1.006	0.9809	1.0193	0.38
A	ENSRNOP00000016432	16	16	91	207	89	75	0.95708	1.02168	0.9835	42	4	3	2	56	8	9	28
RAB2						26.61	6.785				0.991	0.9973		1.0159	1.005	0.9857	1.0144	0.24
3	ENSRNOP00000072716	10	10	37	237	65	64	0.97359	1.00315	0.99684	19	8	1.0031	6	48	9	1	65
RAP2						20.49	4.805				0.980	1.0128	1.0313	1.0057	1.016	0.9645	1.0367	0.22
B	ENSRNOP00000019340	6	9	66	183	12	18	1.00368	1.00487	0.93316	57	2	1	3	62	5	6	14

RAB9B	ENSRNOP00000066233	6	7	19	201	22.704	4.93213	1.08215	1.04237	0.96444	1.02965	0.99636	0.93993	1.01322	0.98317	1.04728	0.95486	0.3209
RAP2C	ENSRNOP00000003414	5	9	46	183	20.7314	4.94482	0.99075	1.00134	1.00484	0.99898	1.00448	0.98585	1.02277	1.00437	0.99463	1.0054	0.6628
RAB39A	ENSRNOP00000011913	7	9	55	217	24.9047	7.94287	1.01662	1.02684	0.95803	1.0005	1.01885	0.9786	0.96458	0.98735	1.01332	0.98686	0.6506
RAN	ENSRNOP00000001247	9	9	55	216	24.4076	7.48877	1.00678	0.99007	0.97016	0.98901	1.0019	1.01976	1.03675	1.01947	0.97012	1.0308	0.1053
RAB4A	ENSRNOP00000048878	3	6	50	218	24.3932	6.07471	1.00308	0.98313	0.95156	0.97926	0.99639	0.97956	1.08617	1.02071	0.95939	1.04233	0.3176
RAB4B	ENSRNOP00000002052	7	10	58	213	23.614	6.06201	0.97895	1.01764	0.98696	0.99452	1.01037	0.98197	0.98775	0.99336	1.00116	0.99884	0.9411
RALA	ENSRNOP00000018190	5	8	72	206	23.538	7.10791	0.96126	1.00666	0.98962	0.98585	1.0318	0.99753	0.97783	1.00239	0.9835	1.01678	0.4668
RAB33A	ENSRNOP00000008868	6	8	16	237	26.5664	7.63525	1.01528	0.99115	0.99639	1.00094	0.97988	1.02901	1.00323	1.00404	0.99691	1.0031	0.8555
RIT2	ENSRNOP00000023871	4	4	5	217	24.7454	6.91748	0.89988	1.08778	1.03233	1.00667	0.97114	0.96254	0.99872	0.97747	1.02987	0.971	0.6343
RHEB	ENSRNOP00000063915	9	9	35	183	20.2814	6.32861	0.99945	1.02848	0.99997	1.0093	1.0109	0.99529	0.98406	0.99675	1.01259	0.98757	0.3671
RAP1B	ENSRNOP00000009511	5	11	77	184	20.7846	5.78271	0.96481	1.03006	0.96466	0.98651	0.99437	1.00228	1.03739	1.01135	0.97544	1.02518	0.3848
RAP1A	ENSRNOP00000040409	5	11	69	184	20.9737	6.66846	1.0178	1.01017	1.00198	1.00998	1.06236	0.93986	0.99943	1.00055	1.00943	0.99066	0.8045
CDC42	ENSRNOP00000018118	2	8	79	191	21.2969	6.03662	0.96677	1.01869	0.98203	0.98916	1.01416	1.01357	1.01358	1.01377	0.97573	1.02488	0.1855

RRAS	ENSRNOP00000027809	5	7	19	218	23.8942	7.34229	1.04129	1.00084	1.02864	1.02359	0.9414	0.87364	0.82523	0.88009	1.16305	0.85981	0.0159
RAB34	ENSRNOP00000036566	5	5	7	255	28.4857	8.08936	1.01545	0.99993	1.00535	1.00691	0.97762	0.9827	1.00139	0.98724	1.01993	0.98046	0.0826
RHOA	ENSRNOP00000066672	4	11	111	193	21.7681	6.1001	0.95266	0.99776	0.97926	0.97656	1.1412	1.12408	1.12182	1.12903	0.86495	1.15613	0.0005
RHOG	ENSRNOP00000068181	8	9	56	185	20.6095	7.95752	0.95597	1.02258	0.97825	0.9856	1.08752	0.92152	0.98091	0.99665	0.98891	1.01121	0.8431
RHOC	ENSRNOP00000017254	1	8	71	193	22.0223	6.58057	0.99616	0.96833	0.98846	0.98432	1.11051	1.16119	1.09146	1.12105	0.87803	1.13892	0.0036
RAC3	ENSRNOP00000064762	3	6	46	192	21.5081	8.14795	1.01831	0.99435	0.94796	0.98687	0.94669	1.00195	1.07223	1.00696	0.98006	1.02035	0.6559
RAC1	ENSRNOP00000001417	5	9	88	192	21.4362	8.49951	0.97147	1.00127	0.94624	0.97299	1.12148	1.13523	1.13194	1.12955	0.8614	1.1609	0.0007
RHOF	ENSRNOP00000063718	8	8	11	211	23.5861	8.42627	1.02296	1.01967	0.98905	1.01056	0.97807	1.01767	0.99683	0.99752	1.01307	0.9871	0.4538
RHOB	ENSRNOP00000008008	8	10	166	196	22.1091	5.23682	1.05993	0.99613	0.98388	1.01331	0.99744	0.98526	0.96776	0.98349	1.03033	0.97057	0.3005
RAB28	ENSRNOP00000050260	3	3	5	220	24.7055	5.46533	1.00589	0.99175	0.98585	0.9945	0.98294	0.97427	1.05696	1.00472	0.98982	1.01028	0.7231
REM1	ENSRNOP00000010149	1	1	1	297	32.8869	9.24658	1.08797	0.99453	0.99097	1.02449	0.98474	0.94521	1.04651	0.99216	1.03259	0.96844	0.4970
RABL3	ENSRNOP00000033847	2	2	2	236	26.2774	7.15186	0.90667	0.9563	1.07905	0.98067	1.05422	1.03345	0.9548	1.01416	0.96698	1.03414	0.6037
REM2	ENSRNOP00000016020	1	1	1	341	37.2519	7.63525	1.2051	0.98583	0.90648	1.03247	0.98363	0.97741	1.02793	0.99632	1.03628	0.96499	0.7106

ARFRP	ENSRNOP00000019038	3	3	6	201	22.6444	6.55127	0.98691	1.01219	0.99557	0.99822	0.98912	1.03904	0.99572	1.00796	0.99034	1.00975	0.6042
ARL8A	ENSRNOP00000008163	2	9	44	186	21.376	7.76709	0.95285	1.03256	0.96194	0.98245	1.00575	1.01859	1.01014	1.01149	0.97129	1.02956	0.3178
ARL8B	ENSRNOP00000071470	3	11	40	186	21.5251	8.42627	0.94038	1.00509	0.97091	0.97213	0.98598	1.05577	1.01732	1.01969	0.95336	1.04892	0.1588
SAR1B	ENSRNOP00000006567	4	7	18	198	22.3955	6.11279	0.93953	1.01727	1.01169	0.9895	0.99078	1.0135	1.01311	1.00579	0.9838	1.01647	0.5667
ARF4	ENSRNOP00000017692	4	9	98	180	20.3836	7.13721	1.03478	0.99264	0.98788	1.0051	1.02019	0.99894	0.98914	1.00276	1.00234	0.99766	0.8997
ARL5A	ENSRNOP00000009181	3	3	3	179	20.7005	6.78564	1.00567	1.01286	0.9657	0.99474	1.00567	1.02701	1.02248	1.01838	0.97679	1.02376	0.2146
ARL1	ENSRNOP00000007623	6	6	15	181	20.3985	5.71924	0.99191	1.00971	1.03924	1.01362	0.97338	1.01782	0.95713	0.98278	1.03138	0.96957	0.2475
ARF6	ENSRNOP00000006355	6	7	26	175	20.0694	8.95361	0.97874	0.98714	0.98791	0.98459	0.98572	1.04819	1.02641	1.02011	0.96519	1.03607	0.1279
ARF5	ENSRNOP00000010429	4	10	152	180	20.5166	6.78564	0.98327	1.00555	1.00795	0.99892	1.00296	1.01711	0.98466	1.00158	0.99735	1.00266	0.8389
ARL15	ENSRNOP00000014761	4	4	15	204	22.8915	5.63037	1.02681	1.01684	0.98811	1.01058	0.98839	1.01296	0.99736	0.99957	1.01102	0.9891	0.4646
ARL6	ENSRNOP00000002293	8	8	21	186	20.9551	8.25049	1.01367	1.02507	0.94967	0.99613	1.01842	0.98622	0.99124	0.99863	0.9975	1.0025	0.9268
ARL3	ENSRNOP00000027093	8	8	27	186	20.853	7.23975	0.96371	1.02193	1.0034	0.99635	0.99721	0.97991	1.01125	0.99612	1.00023	0.99978	0.9913
ARL2	ENSRNOP00000028525	5	5	12	175	19.8532	5.42725	1.00012	1.04186	0.98154	1.00784	0.98393	1.01088	0.98344	0.99275	1.0152	0.98503	0.4926

ARL10	ENSRNOP00000066910	1	1	2	193	21.6182	4.80518	0.98112	1.01823	0.97197	0.99044	0.96022	1.0196	1.05323	1.01102	0.97965	1.02078	0.5388

Supplementary Table S3. Key resources table

Resource Type	Specific Reagent or Resource	Source or Reference	Identifiers	Additional Information
Antibody	rabbit anti-SK2 monoclonal antibody	Alomone, Jerusalem, Israel	Cat.# APC-045	
Antibody	rabbit anti-SK3 monoclonal antibody	Alomone, Jerusalem, Israel	Cat.# APC-025	
Antibody	mouse monoclonal anti-NeuN	Millipore, Billerica, USA	Cat.# MAB377	
Reagent	tetramethylrhodamine-dextran, (TMR,3,000 MW)	Molecular Probe, Eugene, OR	Cat.# D3308	
Antibody	Rabbit anti-Casein Kinase II α polyclonal antibody	Sigma-Aldrich	Cat.# SAB4500514	
Antibody	Rabbit anti-Casein Kinase II β polyclonal antibody	Sigma-Aldrich	Cat.# SAB4500516	

Chemical Compound or Drug	NSC23766	Abcam	Cat.# ab142161
Antibody	Rabbit anti-PP2A polyclonal antibody	Sigma-Aldrich	Cat.# SAB4502298
Commercial Assay Or Kit	Active Rac1 Pull-Down and Detection Kit	Thermo Scientific	Cat.# 16118
Antibody	Rabbit anti-β-actin antibody	ZSGB-BIO Co., Beijing, China	Cat.# TA-09
Antibody	Rabbit anti-β-Tubulin antibody	Sigma-Aldrich	Cat.# SAB4500088
Antibody	Rabbit anti-TMR monoclonal antibody	Invitrogen, United States	Cat.# A-6397
Antibody	Cy2-conjugated goat anti-mouse IgG	Jackson Immunoresearch	Cat.# 115-225-071

Antibody	Cy3-AffiniPure goat anti-rabbit	Jackson Immunoresearch	Cat.# 111-095-003	
Reagent	4',6-Diamidino-2-phenylindole dihydrochloride (DAPI)	Sigma-Aldrich	Cat.# D9542	
Chemical Compound or Drug	Apamin	Sigma-Aldrich	Cat.# A1289	
Genetic Reagent	Lentivirus expressing Rac1-shRNAs-green fluorescent protein	Genepharma Technology Co., Ltd (Shanghai, China)	LV3(H1/GFP&Puro)	The target shRNA regions were chosen as follows: Rac1-124, GCCAATGTTATGGTAGATGGA; Rac1-219, GCAAACAGACGTGTTCTTAAT; Rac1-340, GGGACGAAGCTTGATCTTAGG; negative control, TTCTCCGAACGTGTCACGT.
Software; Algorithm	Prism 8.1.2 software	Graphpad		
Software; Algorithm	Origin 9.0	OriginLab		

Supplementary References

1. Qu L, Wang Y, Ge SN, Li N, Fu J, Zhang Y, et al. Altered Activity of SK Channel Underpins Morphine Withdrawal Relevant Psychiatric Deficiency in Infralimbic to Accumbens Shell Pathway. Frontiers in psychiatry. 2019; 10: 240.
2. Hopf FW, Bowers MS, Chang SJ, Chen BT, Martin M, Seif T, et al. Reduced nucleus accumbens SK channel activity enhances alcohol seeking during abstinence. Neuron. 2010; 65: 682-94.
3. Wang XQ, Ma J, Cui W, Yuan WX, Zhu G, Yang Q, et al. The endocannabinoid system regulates synaptic transmission in nucleus accumbens by increasing DAGL-alpha expression following short-term morphine withdrawal. British journal of pharmacology. 2016; 173: 1143-53.
4. Bracci E, Centonze D, Bernardi G, Calabresi P. Dopamine excites fast-spiking interneurons in the striatum. Journal of neurophysiology. 2002; 87: 2190-4.
5. Klenowski PM, Shariff MR, Belmer A, Fogarty MJ, Mu EW, Bellingham MC, et al. Prolonged Consumption of Sucrose in a Binge-Like Manner, Alters the Morphology of Medium Spiny Neurons in the Nucleus Accumbens Shell. Front Behav Neurosci. 2016; 10: 54.
6. Bennett BD, Callaway JC, Wilson CJ. Intrinsic membrane properties underlying spontaneous tonic firing in neostriatal cholinergic interneurons. The Journal of neuroscience : the official journal of the Society for Neuroscience. 2000; 20: 8493-503.
7. Mi Z, Si T, Kapadia K, Li Q, Muma NA. Receptor-stimulated transamidation induces activation of Rac1 and Cdc42 and the regulation of dendritic spines. Neuropharmacology. 2017; 117: 93-105.
8. Fakira AK, Portugal GS, Carusillo B, Melyan Z, Moron JA. Increased small conductance calcium-activated potassium type 2 channel-mediated negative feedback on N-methyl-D-aspartate receptors impairs synaptic plasticity following context-dependent sensitization to morphine. Biological psychiatry. 2014; 75: 105-14.
9. Du TT, Chen YC, Lu YQ, Meng FG, Yang H, Zhang JG. Subthalamic nucleus deep brain stimulation protects neurons by activating autophagy via PP2A inactivation in a rat model of Parkinson's disease. Experimental neurology. 2018; 306: 232-42.
10. Reissner KJ, Uys JD, Schwacke JH, Comte-Walters S, Rutherford-Bethard JL, Dunn TE, et al. AKAP signaling in reinstated cocaine seeking revealed by iTRAQ proteomic analysis. The Journal of neuroscience : the official journal of the Society for Neuroscience. 2011; 31: 5648-58.
11. Zhu B, Li X, Chen H, Wang H, Zhu X, Hou H, et al. iTRAQ proteomic analysis of the hippocampus in a rat model of nicotine-induced conditioned place preference. Biochemical and biophysical research communications. 2017; 486: 971-7.
12. Wang W, Ju YY, Zhou QX, Tang JX, Li M, Zhang L, et al. The Small GTPase Rac1 Contributes to Extinction of Aversive Memories of Drug Withdrawal by Facilitating GABAA Receptor Endocytosis in the vmPFC. The Journal of neuroscience : the official journal of the Society for Neuroscience. 2017; 37: 7096-110.
13. Royo NC, Vandenberghe LH, Ma JY, Hauspurg A, Yu L, Maronski M, et al. Specific AAV serotypes stably transduce primary hippocampal and cortical cultures with high efficiency and low toxicity. Brain research. 2008; 1190: 15-22.
14. Khalil-Khalili M, Rashidy-Pour A, Bandegi AR, Yousefi B, Jorjani H, Miladi-Gorji H. Effects of BDNF receptor antagonist on the severity of physical and psychological dependence, morphine-induced locomotor sensitization and the ventral tegmental area-nucleus accumbens BDNF levels in morphine-dependent and withdrawn rats. Neuroscience letters. 2018; 668: 7-12.
